# Self-driving bioprinting laboratories

**DOI:** 10.1088/1758-5090/ae3645

**Published:** 2026-01-22

**Authors:** Suihong Liu, Navneet Kaur, Dae-Hyeon Song, Joseph Christakiran Moses, Ibrahim T Ozbolat

**Affiliations:** 1 The Huck Institutes of Life Sciences, Penn State University, University Park, State College, PA 16802, United States of America; 2 Engineering Science and Mechanics Department, Penn State University, University Park, State College, PA 16802, United States of America; 3 Biomedical Engineering Department, Penn State University, University Park, State College, PA 16802, United States of America; 4 Materials Research Institute, Penn State University, University Park, State College, PA 16802, United States of America; 5 Cancer Institute, Penn State University, University Park, State College, PA 16802, United States of America; 6 Neurosurgery Department, Penn State University Hershey, Hershey, PA 17033, United States of America

**Keywords:** self-driving laboratories, artificial intelligence, bioprinting, robotics, bioreactor

## Abstract

The severe shortage of donor organs and limitations of current disease models highlight the urgent need for transformative strategies in tissue engineering (TE) and regenerative medicine (RM). Bioprinting has emerged as a powerful approach for creating functional tissues and organs, yet current workflows remain labor-intensive, variable, and challenging to scale. The convergence of artificial intelligence (AI), advanced bioprinting technologies, robotics, biosensing, and cutting-edge biological methods is catalyzing the development of self-driving bioprinting laboratories—a fully integrated, autonomous, closed-loop system capable of designing, fabricating, maturing, and assessing living tissue constructs, as well as supporting seamless transplantation, with minimal human intervention. By integrating autonomous cellular farming, on-demand bioink formulation, intelligent optical and digital reconstruction platforms, AI-driven bioprinting, intelligent bioreactors, and robotic transplantation within a sterile, interconnected ecosystem, such platforms can continuously learn, adapt, and optimize workflows, enabling standardized, scalable tissue manufacturing and facilitating a seamless transition from bench to bedside. This perspective outlines the foundational technologies, opportunities, and challenges for realizing self-driving bioprinting, envisioning a future where intelligent, automated platforms transform TE and RM into a scalable, predictive, and clinically integrated discipline at the forefront of precision medicine.

## 1. Introduction

The escalating demand for organ repair and transplantation, coupled with the severe shortage of available donors, underscores the urgent clinical need to develop innovative treatment strategies for repair and regeneration of damaged tissues and organs [1]. Additionally, disease research and drug development still heavily rely on two-dimensional (2D) cell models and animal models, both of which cannot accurately mimic and replicate the complex physiological environment in humans, meanwhile resulting in protracted development cycles, high costs, and failure rates [2]. In this context, tissue engineering (TE) and regenerative medicine (RM) present promising solutions to mitigate the challenges associated with organ transplantation and drug development, particularly through the integration of rapidly advancing three-dimensional (3D) bioprinting technologies [3–5]. Bioprinting originated with the pioneering work of Klebe in 1998 [6], who introduced cytoscribing technology as a method for 2D micropositioning of proteins. The explicit term ‘bioprinting’ began to appear consistently in the literatures around 2004 [7], marking the point at which the field started to coalesce conceptually and technologically. Beginning around 2008, bioprinting entered a phase of accelerated expansion. Over the past two decades, bioprinting has rapidly evolved into a highly interdisciplinary field integrating biology, biomaterials, engineering, and computational technologies. Today, bioprinting enables the precise spatial organization of cells, biomaterials, and bioactive factors to fabricate biomimetic, living 3D constructs across multiple scales, achieving resolutions down to the level of individual cells [8, 9]. These advances nowadays enable the fabrication of increasingly biomimetic and functional constructs, underscoring bioprinting’s growing potential in TE, RM, and translational biomedical applications.

Various bioprinting technologies have been explored in recent years, including extrusion-[10], droplet- [11], laser- [12], light- [13–15], and acoustic-based bioprinting [16, 17]. Each of these techniques offers unique advantages in terms of printing speed and resolution, cell viability, and material compatibility, enabling various applications. Bioprinting inherently requires a bioink, which was defined in 2019 [18] as ‘*a formulation of cells suitable for processing by an automated biofabrication technology that may also contain biologically active components and biomaterials*.’ The design and application of bioinks have significantly expanded over the past decades, with a wide variety of materials—primarily natural and synthetic hydrogels, being developed and adapted to meet the stringent demands of bioprinting [19–22]. Recognizing the rapid advancements and growing interest in the field, the biofabrication community introduced a comprehensive bioprinting roadmap in 2020 [5], which highlighted key achievements, ongoing challenges, and future scientific and technological goals for this evolving discipline. Nowadays, while new bioprinting techniques continue to evolve, the principles and mechanisms of bioprinting are well-established and extensively validated. Most of the current research primarily focuses on optimizing bioink formulations, enhancing bioprinting precision, stability, and reproducibility, advancing the construction of functional tissue and organ analogs, and expediting the translation of these technologies and products into clinical applications.

The bioprinting process can be broadly divided into three interconnected stages: pre-bioprinting, bioprinting, and post-bioprinting, each influencing and constrained by the others. In the pre-bioprinting stage, efforts focus on the development and optimization of bioinks, which form the foundation of tissue constructs [23]. This involves synthesizing and modifying bioinks to meet biological and bioprinting requirements, alongside acquiring, expanding, and conditioning cells to ensure viability and functionality. Although these procedures are well-established and standardized, they remain labor-intensive and time-consuming, creating operational bottlenecks [24]. Similar challenges persist in the bioprinting and post-bioprinting stages. During bioprinting, tasks such as achieving bioprinting precision, construct reproducibility, and throughput optimization often require repetitive interventions. Post-bioprinting further demands tissue maturation, functional validation, and the maintenance of tightly controlled environments to preserve viability and functionality, particularly for clinical translation.

To overcome these challenges, automating and optimizing each stage of bioprinting is critical. Recent studies highlight the integration of robotics, artificial intelligence (AI), and advanced biosensing techniques as promising strategies to enhance efficiency, precision, and reproducibility [25]. Building on these innovations, the development of intelligent, fully automated ‘self-driving’ bioprinting laboratories—seamlessly integrating these tools within a unified workflow—promises to usher in a new era in TE and RM. The ultimate goal of these laboratories is to revolutionize TE and RM by streamlining processes, improving precision, reducing costs, and accelerating the clinical translation of engineered tissues. By standardizing complex bioprinting procedures and enabling high-throughput, patient-specific tissue production through real-time monitoring, adaptive control systems, and predictive AI-driven analytics, self-driving laboratories could transform TM and RM from labor-intensive, experimental practices into reproducible, scalable, and clinically viable therapies. This paradigm shift would bridge the gap between laboratory innovation and clinical application, positioning automation and intelligent systems as central drivers of the future of TE and RM. This perspective highlights how the convergence of automation, AI, and advanced bioprinting technologies offers a promising pathway toward this transformative vision.

## 2. Needs and challenges in bioprinting

**Figure 1.**
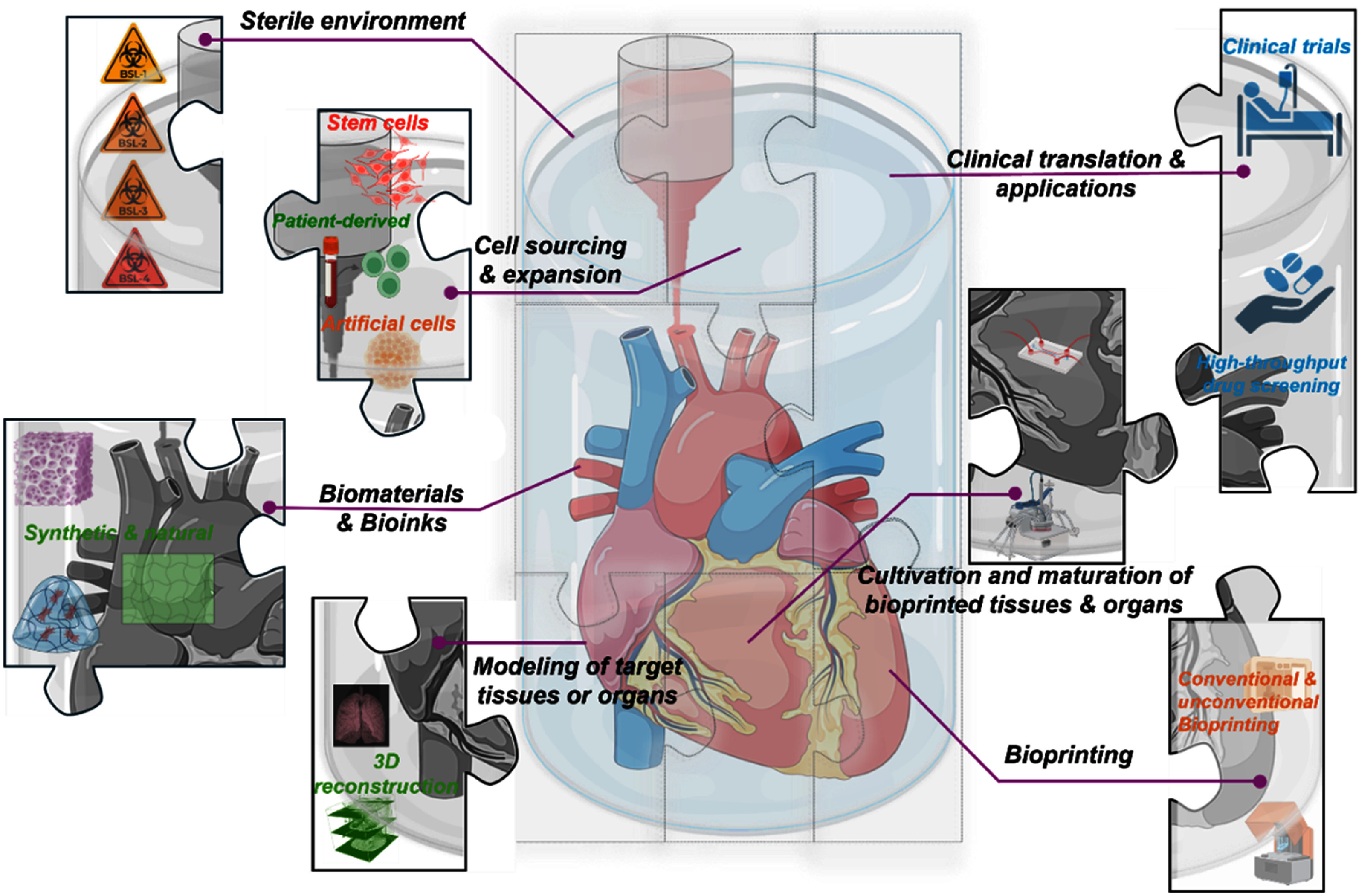
Key components and strategies for bioprinting of functional tissues and organs. Created in BioRender. Ozbolat, I. (2026) https://BioRender.com/ qwaky4z

Bioprinting is a multifaceted process that requires the integration of diverse critical elements to engineer functional tissues and organs [26]. As illustrated in figure 1, these elements can be broadly categorized into seven interdependent domains: a controlled sterile environment, robust and scalable cell sourcing, well-designed biomaterials and bioinks, advanced scanning and computational modeling, scalable bioprinting processes, intelligent bioreactors for tissue maturation, and strategies for clinical translation. Together, these interconnected domains form the essential foundation for advancing bioprinting and driving its successful translation from laboratory research to clinical applications. Achieving this goal requires the development of intelligent, automated systems capable of seamlessly integrating, monitoring, and controlling each component to ensure standardized, efficient, and reproducible processes.

### 2.1. A sterile environment

Maintaining a sterile environment is essential across all stages of the bioprinting workflow, including material preparation, bioink formulation, cell culture, bioprinting, post-bioprinting handling, *in vitro* tissue maturation, and *in vivo* implantation. Each stage should take place in dedicated sterile zones equipped with advanced sterilization systems to prevent contamination and safeguard the integrity of bioprinting processes. These zones must be interconnected through secure, sterilized transport systems, such as automated robotic carriers or enclosed pathways, ensuring safe and contamination-free material and construct transfer. Whole lab should be equipped with sterilized instruments, consumables, and state-of-the-art monitoring systems [27] to continuously assess and verify environmental sterility, including air cleanliness, particulate levels, and microbial presence. Integrated real-time feedback mechanisms should promptly detect deviations from sterility and enable immediate corrective actions. Adhering to these stringent standards ensures the production of high-quality, safe, and functional bioprinted tissues and organs suitable for research and clinical applications. Importantly, the establishment and maintenance of sterile, tightly controlled cleanroom environments has already been successfully implemented in many industrial biofabrication settings functioning under Good Manufacturing Practice (GMP) frameworks [27], providing a proven foundation for the future development of self-driving bioprinting laboratories.

### 2.2. Cell sourcing and expansion

Cell sourcing and expansion are critical challenges in bioprinting, as replicating the high cellular density of natural tissues and organs requires a substantial number of cells [4, 28]. For instant, reported hepatocyte counts for adult human liver range from 8 × 10^10^ to 2.5 × 10^11^ cells [29, 30]. Assuming a representative confluent harvest of ∼3.5 × 10^6^ cells per T-175 flask, generating this number of hepatocytes would require approximately 2.3 × 10_⁴_ to 7.1 × 10_⁴_ T-175 flasks. Given that a standard large-capacity CO_₂_ incubator typically accommodates 120-200 T-175 flasks, this expansion would necessitate approximately 160–590 incubators operating in parallel. Beyond the sheer magnitude of cell numbers, the diversity of required cell types further amplifies the challenge. Recent single-cell and spatial transcriptomics studies estimate that the adult human body contains at least 200–400 distinct cell types, each with unique phenotypes, microenvironmental requirements, and expansion constraints [31, 32]. This cellular heterogeneity underscores the immense complexity of sourcing, expanding, and maintaining physiologically relevant cell populations for large-scale bioprinting.

Current cell sources mainly include primary, stem, and progenitor cells. Primary cells, directly harvested from donor tissues, offer physiological relevance but are limited by scarce availability and restricted expansion potential. Stem cells—such as embryonic stem cells (ESCs), mesenchymal stem cells (MSCs), and induced pluripotent stem cells (iPSCs)—provide broader versatility, with iPSCs enabling patient-specific applications through differentiation into diverse cell types. Progenitor cells, being partially differentiated, balance multipotency with functional specificity. However, scaling up the production of these cells remains a significant challenge [33, 34]. Recently, artificial cells [35], engineered to mimic the structure and functions of natural cells, have advanced significantly and show great promise as an alternative, scalable cellular source for future bioprinting. Traditional 2D culture systems are inadequate for large-scale expansion, leading to the development of advanced methods such as microcarrier-based 3D culture platforms, bioreactors, and organ-on-a-chip systems [36]. Bioreactors provide a controlled environment with precise regulation of oxygen, nutrients, and waste, while microcarrier systems enhance scalability by offering a larger surface area for cell growth [37]. Dynamic culture systems further promote efficient expansion by simulating physiological conditions through mechanical and biochemical cues. Addressing the complexities of cell sourcing and developing robust, scalable culture systems are essential to ensure a reliable supply of high-quality cells for bioprinting functional tissues and organs.

### 2.3. Biomaterials and bioinks

In bioprinting, selecting appropriate biomaterials and bioinks is critical for regenerating human tissues and organs [38]. These biomaterials must possess high biocompatibility, mechanical strength, biodegradability, and the ability to support cell growth and differentiation. Commonly used biomaterials comprise both natural and synthetic biopolymers, sourced from a wide range of origins, including animals, humans, plants, marine organisms, bacteria, and other microorganisms. Each biomaterial type offers distinct benefits and limitations depending on the application [39]. Bioinks, which combine biomaterials with cellular building blocks (cells, spheroids, organoids, etc.), are essential for 3D bioprinting of tissues, but developing the ideal bioink is a complex and time-consuming process. These bioinks must balance printability, biological functionality, and mechanical integrity after bioprinting, a challenge that makes the development process both iterative and lengthy [40]. Printability can be compromised by suboptimal rheological properties (e.g. viscosity, shear-thinning behavior, yield stress, and crosslinking kinetics), leading to poor filament formation, extrusion instability, or low geometric fidelity in the context of extrusion-based bioprinting. Biological functionality is influenced by cytocompatibility, biochemical signaling cues, biodegradability, and the capacity to support cell adhesion, proliferation, and differentiation, all of which vary across biomaterial systems and cell types. Mechanical integrity after printing depends on polymer concentration, crosslinking density, and network architecture, which determine both the immediate structural stability of constructs and their long-term behavior under physiological loading. These factors often exhibit non-linear trade-offs—for instance, increasing viscosity or crosslinking may enhance shape retention but impair cell viability or tissue remodeling—representing major challenges for current bioink development. Furthermore, large-scale production of bioinks remains costly, as it involves intricate biomaterial extraction, purification, and testing. Therefore, integrating AI and automation into bioink development and synthesis represents a pivotal step toward advancing bioprinting into clinically viable applications. An AI-driven bioink platform could systematically explore and optimize the multidimensional biomaterial design space to generate tissue-specific formulations on demand, while ensuring batch-to-batch consistency, physicochemical stability, and high-throughput production at clinically relevant scales. By automating iterative optimization cycles, reducing dependence on manual labor, and enabling the transition from milliliter-scale laboratory preparation to liter-scale manufacturing, such intelligent systems have the potential to overcome major bottlenecks in contemporary bioink engineering. Collectively, these capabilities would create a more robust, reproducible, and scalable foundation for the clinical translation and widespread deployment of bioprinted tissues and organs.

### 2.4. Tissue modeling and fidelity

In bioprinting, blueprint modeling of tissues or organs, particularly those with defects, is a fundamental step in creating functional replacements [26, 41]. However, the inherent complexity and multi-scale organization of human tissues—from subcellular structures to organ-level architecture—pose significant challenges. Many tissue types remain poorly characterized at the nano-, micro-, and mesoscale, and the dynamic interactions between cells, extracellular matrices (ECM), and vascular networks are not fully understood. Understanding these structural and functional intricacies is essential before embarking on the design, build, and commissioning of a bioprinting system, as it directly affects the fidelity, functionality, and maturation of the final construct. Imaging techniques such as computed tomography (CT) and magnetic resonance imaging (MRI) are essential for capturing detailed anatomical data, which are then processed into digital 3D models. These models serve as blueprints for bioprinting, and their precision directly impacts the quality and functionality of the final tissue or organ. Establishing a seamless relationship between the reconstructed model and the bioprinting process is critical, especially when considering the varying resolutions of different bioprinting technologies. The model resolution needs to align with the bioprinting capabilities to ensure the final tissue structure is accurately replicated. The model-building process is inherently iterative, requiring continuous refinement to ensure compatibility with the specific requirements of the bioprinting technology, such as resolution, biomaterial compatibility, and structural integrity. Current modeling methods primarily rely on converting imaging data into 3D digital formats, followed by computational simulations to adjust the models for bioprinting processes [42]. However, several challenges persist, such as the mismatch between model resolution and the bioprinting technology’s resolution [43], difficulties in capturing complex tissue architectures (like vascular networks and cellular arrangements), and integrating multiple bioinks to replicate the heterogeneity of natural tissues [39]. Overcoming these challenges will require AI-assisted knowledge systems capable of multiscale tissue and organ understanding, alongside continued advancements in imaging modalities, computational modeling, and bioprinting platforms. These improvements are crucial for enhancing the accuracy, complexity, and functionality of tissues and organs, moving us closer to their clinical application.

### 2.5. Bioprinting

In 3D bioprinting, two primary approaches are utilized: *in vitro* and *in situ (or in vivo)*. In the former, bioprinting of tissues or organs is performed outside the patient’s body, followed by culture, allowing their maturation *in vitro* before transplantation. In contrast, *in vivo* bioprinting involves direct deposition of bioink into the recipient’s body, facilitating real-time integration with existing tissues without external culture or implantation surgery, offering potential for seamless tissue regeneration. *In vitro* bioprinting utilizes diverse bioprinting technologies to precisely deposit cells and biomaterials for constructing complex tissue constructs. While these methods have enabled significant progress, they face challenges in maintaining cell viability, achieving appropriate mechanical properties, and establishing functional vascular networks [44]. *In vivo* bioprinting, on the other hand, directly deposits bioinks within the recipient’s body to enable *in situ* tissue regeneration, minimizing the need for external culture and implantation [45]. Current approaches include laser-based, extrusion-based, ultrasound-assisted, and light-based bioprinting techniques, each offering unique advantages, such as targeted material placement, continuous bioink delivery, non-invasive cell positioning, and high-resolution crosslinking within living tissues [46]. Despite its promise, *in vivo* bioprinting remains limited by difficulties in preserving cell viability, controlling deposition within dynamic tissues, and achieving durable structural and functional integration. The required complexity of creating multi-cellular, vascularized and innervated tissues further heightens these challenges, underscoring the need for continued innovation in biomaterials, bioprinting technologies, and biological strategies. To date, bioprinting’s clinical impact is limited by variability, low reproducibility, and technical constraints such as resolution and scalability. Self-driving bioprinting system could improve repeatability, robustness, and process standardization through AI-driven decision-making, real-time monitoring, and closed-loop control. However, automation alone cannot overcome inherent technological limits—for example, AI cannot increase the resolution of a low-precision bioprinter. Achieving clinically relevant tissues will therefore require high-performance hardware combined with intelligent, scalable, and adaptive bioprinting platforms capable of producing functional, multicellular, and vascularized constructs.

### 2.6. Cultivation and maturation of bioprinted tissues and organs

The cultivation and maturation of bioprinted tissues and organs are essential for their functional integration into the human body. After *in vitro* bioprinting, tissues or organs are needed to be cultured in incubators or bioreactors where they receive nutrients, oxygen, and growth factors to promote cellular growth and tissue remodeling. Bioreactors are designed to mimic the human body’s supply systems, utilizing perfusion mechanisms to simulate blood circulation, enhancing nutrient and oxygen delivery, and facilitating waste removal [47]. Mechanical, electrical, or biochemical stimulations, such as cyclic strain, shear stress, and electrical fields, are applied to promote tissue maturation, cellular alignment, and ECM formation [48]. Recent advancements focus on optimizing bioreactor designs to more closely replicate the physiological environment. For instance, 3D-printed perfusion bioreactors have been developed to provide continuous nutrient flow, ensuring uniform distribution across tissue constructs, while integrating sensors for real-time monitoring of parameters like pH, temperature, and oxygen levels [36, 49, 50]. These technologies are crucial for assessing tissue health and maturation, as fluctuations in these parameters can significantly affect cellular behavior. Despite these advances, challenges persist, particularly in achieving effective vascularization, which is critical for supplying nutrients to larger constructs and preventing necrosis [51]. Approaches such as use of sacrificial materials to create vascular channels and applying mechanical and electrical stimuli during culture are being explored to address these issues. For example, cyclic mechanical loading can enhance cellular alignment and ECM deposition, while electrical stimulation influences cell differentiation and tissue organization. These advancements in bioreactor design, sensor integration, and stimulation techniques are addressing key challenges and moving the field closer to clinical applications. However, our understanding of the regulatory mechanisms underlying human tissue and organ development—including growth, differentiation, and response to dynamic stimuli—remains incomplete. As a result, constructing standalone tissue- or organ-scale bioreactors capable of faithfully recapitulating complex organ functions is extremely challenging. In addition, optimal *in vitro* culture durations, as well as standardized protocols for real-time monitoring, functional assessment, and dynamic stimulation are not yet well defined. To address these limitations, AI-driven adaptive bioreactors that can emulate key aspects of human physiology hold significant promises for accelerating tissue maturation, enhancing reproducibility, and facilitating the clinical translation of engineered tissues.

### 2.7. Clinical translation and applications

The clinical translation of bioprinted tissues and organs involves several critical steps, including transplantation through surgical procedures, with challenges related to maintaining tissue viability during transplantation and ensuring proper integration with the host tissue. Modular handheld bioprinters have been developed as flexible intraoperative tools, enabling controlled deposition of multiple biomaterials and cell types with defined geometries and compositions, offering promising potential for clinical applications [52]. However, manual operation inherently limits spatial precision, reproducibility, and process stability, and outcomes remain highly dependent on the operator’s skill and experience. Robotic-assisted surgery offers promising solutions, enhancing precision and control during transplantation, potentially improving outcomes and reducing recovery times [53] additionally, robotic-assisted automated *in vivo* bioprinting, which directly deposits biological materials within the recipient’s body, is being explored to facilitate tissue regeneration and repair. However, ensuring the long-term survival and function of transplanted tissues and organs remains a significant challenge, with strategies focusing on optimizing constructs for vascularization and integration with host tissues, as well as developing methods to monitor and support tissue health post transplantation. Immunological considerations are also crucial, as the immune response can lead to rejection or inflammation [54]. Using patient-specific cells or autologous tissues can mitigate immune rejection, but this approach may not always be feasible. Therefore, developing immunologically compatible biomaterials and incorporating immunomodulatory factors into the fabricated constructs is essential. Additionally, engraftment of bioprinted tissues with surrounding tissues is vital, requiring precise control over mechanical properties during bioprinting to match the host tissue’s strength and function [55]. The dynamic mechanical environment within the body, including shear stress and cyclic loading, must also be accounted for during the design and maturation phases. Scalability and reproducibility of bioprinting processes are also significant hurdles, necessitating standardized protocols for quality control (QC) and regulatory frameworks to ensure consistent outcomes. In summary, clinical translation of bioprinted tissues faces multifaceted challenges related to surgical procedures, immunological compatibility, mechanical integration, dynamic monitoring and standardization. Overcoming these challenges through interdisciplinary research and technological advancements is essential for the successful implementation of bioprinted tissues and organs in clinical settings.

## 3. Self-driving laboratories in bioprinting

The convergence of AI, robotics, bioprinting, and biosensing is paving the way for the next frontiers in RM: self-driving bioprinting laboratories. Envisioned as next-generation intelligent and fully automated platforms, such laboratories are expected to operate under closed-loop control—leveraging real-time data acquisition, machine learning, and robotic execution—to eventually enable autonomous design, fabrication, maturation, and evaluation of biological tissues and organs. In contrast to traditional laboratories, which are limited by manual throughput, variability, and scalability challenges, the self-driving bioprinting laboratories aspire to emulate the efficiency of autonomous systems already transforming fields such as materials science and synthetic biology. To distinguish them from traditional automated or semi-intelligent laboratories that lack integrated real-time decision-making, adaptive optimization, and self-improving capabilities, we propose that self-driving bioprinting laboratories should be defined as ‘*integrated, autonomous, closed-loop bioprinting systems that continuously sense, analyze, decide, and learn—using AI-driven predictive and adaptive control—to independently design, execute, and iteratively optimize tissue fabrication, mature, and translational workflows end to end*’. Drawing from recent advances in automated experimentation, digital twins, and AI-driven optimization, such laboratories integrate all core bioprinting components into a single sterile, networked ecosystem that can operate continuously, learn from past outcomes, and iteratively improve its performance—transforming TE into a data-rich, predictive, and scalable production.

Building on the essential components outlined in section 2, realizing this vision requires the evolution of several key subsystems into intelligent, autonomous modules. First, ***self-driving cell culture and expansion systems*** must be capable of autonomously monitoring cell health, growth kinetics, and phenotype through high-content imaging and multi-omics data, while dynamically adjusting culture parameters via reinforcement learning and predictive models. Second, ***automated bioink synthesis platforms*** should formulate highly customized, functionally-graded bioinks on demand, integrating real-time physicochemical sensing and digital rheology to ensure reproducibility and performance tailored to specific applications. Third, ***intelligent bio-optical and digital reconstruction center*** should acquire high-fidelity data of patient-specific defects and target tissues, generate accurate digital models, and automatically convert these models into bioprinting instructions tailored to different bioprinting modalities and printhead configurations. Fourth, ***AI-guided self-driving bioprinting*** units must translate digital tissue blueprints into complex constructs using multi-cellular, multi-material deposition, *in-situ* monitoring, and feedback-driven error correction. Fifth, ***intelligent bioreactor systems*** should simulate native tissue environments through closed-loop control of biochemical, mechanical, and electrical cues—guided by digital twins and non-destructive biosensor real-time monitoring and feedback—to direct tissue maturation. Sixth, ***autonomous transplantation and clinical translation modules*** will integrate surgical robotics, patient-specific data, and regulatory-compliant automation to enable seamless transition from bench to bedside.

**Figure 2.**
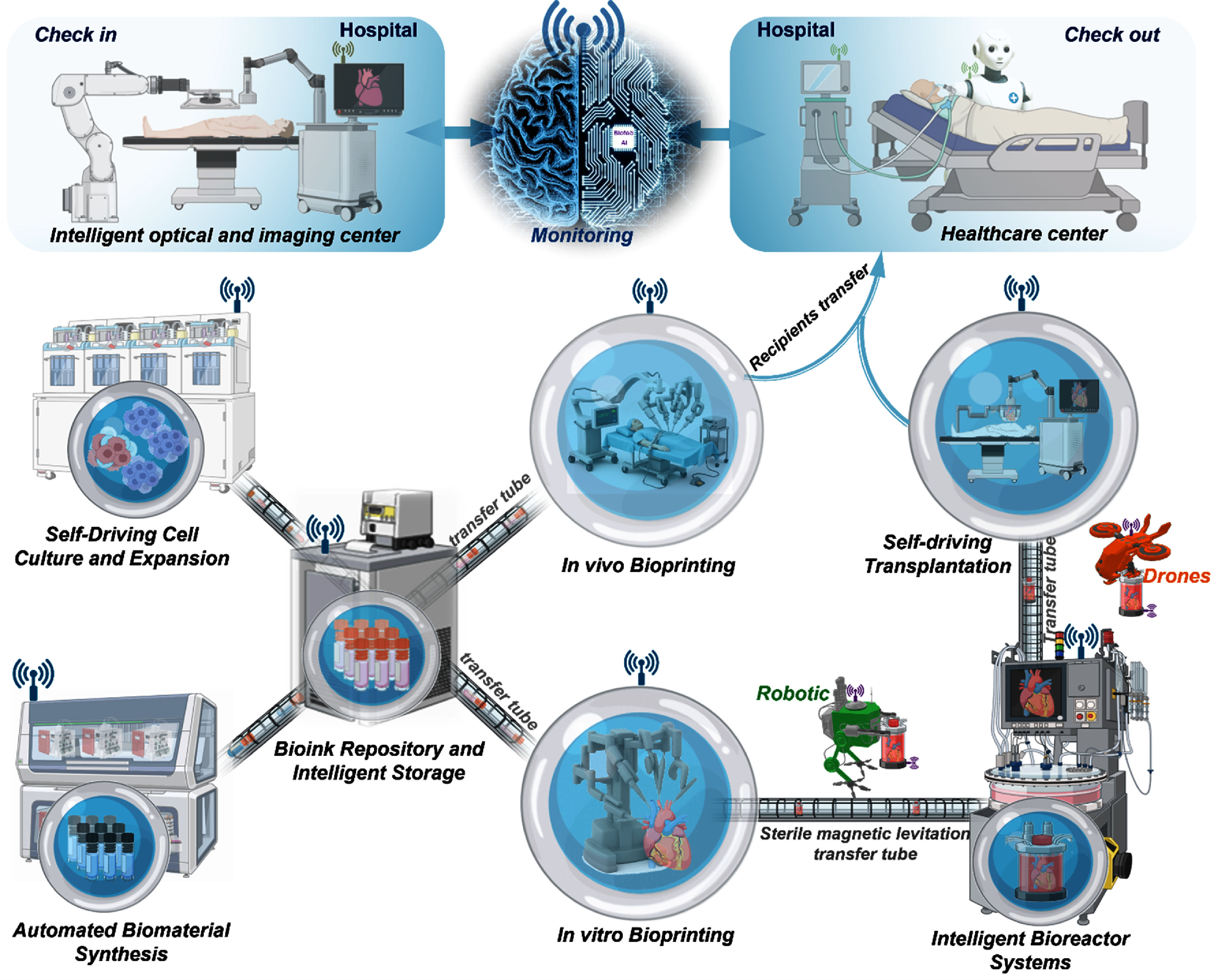
Components and workflow of self-driving bioprinting laboratories. Created in BioRender. Ozbolat, I. (2026) https://BioRender.com/ zqkq3ng

Importantly, all these modules must be physically and digitally interconnected through sterile robotic systems, automated logistics networks, and cloud-based data infrastructures to enable seamless communication and coordination, as illustrated in figure 2. The laboratories should function as an integrated cyber-physical system in which AI can autonomously make critical decisions—such as adjusting pH, modifying print paths, or switching bioinks—in response to a dynamic biological context, while allowing for human oversight or simpler control interventions where appropriate. As these systems evolve, autonomy can progressively increase, complementing human input and enhancing overall process optimization.

Looking ahead, self-driving bioprinting laboratories are not merely a vision of efficiency—they are a transformative model that could unlock truly personalized regenerative therapies, accelerate discovery cycles from years to days, and democratize access to complex tissue products. Such systems could one day be deployed in hospitals or remote clinics, automatically bioprinting skin grafts, vascular patches, or organoids using patient-derived cells. In the long term, this approach may redefine what it means to engineer life, establishing a new paradigm where machines and biology co-evolve—not just to replicate organs, but to design entirely novel therapeutic systems that transcend natural evolution. While significant challenges remain in terms of regulatory acceptance, standardization, and ethical governance, the trajectory is clear: the self-driving bioprinting laboratories will be central to the future of precision medicine, living therapeutics, and the next generation of human health technologies.

### 3.1. Self-driving cell culture and expansion

In the envisioned self-driving bioprinting laboratories, the autonomous sourcing and expansion of cells will serve as a foundational subsystem. Given that cells are the essential living component of any engineered tissue or organ, their controlled production—both in quantity and quality—is vital to the overall success of bioprinting [5]. A critical first step is cell sourcing, which will need to be fully integrated into an intelligent, automated pipeline. A wide array of sourcing strategy is currently used, including primary patient-derived cells, established immortalized cell lines, various stem cell types, and more recently, reprogrammed or synthetic cells [35]. Each strategy offers distinct advantages and limitations that must be considered when building future self-driving bioprinting laboratories.

Primary cells derived from patient biopsies are highly relevant for autologous applications and personalized medicine, as they carry native phenotypes and immune profiles. However, their restricted proliferative capacity, stochastic growth behavior, pronounced donor-to-donor heterogeneity, and susceptibility to senescence substantially limit their suitability for high-throughput, large-scale, or long-term manufacturing workflows. Immortalized cell lines, such as HEK293 or C2C12, offer the benefit of indefinite expansion and experimental reproducibility, yet their often-altered genotypes and phenotypes make them less suitable for clinical applications [56] Stem cells, particularly MSCs, offer immunomodulatory potential and multipotency, though their differentiation range and scalability may decline with repeated passage. ESCs, despite their pluripotency, face ethical and regulatory barriers. iPSCs stand out as a flexible and ethically acceptable alternative, offering patient-specificity and pluripotency. However, iPSCs are still limited by high production costs, risks of tumorigenicity, and complex QC requirements [57] Emerging strategies, such as transdifferentiation and direct lineage reprogramming, are promising for generating target cell types on-demand without reverting to a pluripotent state but remain in early development [58].

Realizing a fully autonomous bioprinting pipeline requires the seamless integration of cell sources into scalable, self-regulating expansion modules. Contemporary progress in bioreactor engineering and culture automation highlights the potential to bring this paradigm to fruition [59, 60] AI-integrated systems, such as CellXpress.ai [61] and other smart incubators, are now capable of monitoring cell confluency, morphology, and health metrics in real time through integrated imaging and sensor data, allowing for dynamic optimization of media exchange, passaging schedules, and environmental control. These platforms reduce the need for manual intervention and enable reproducibility at scale. Closed-system bioreactors—such as hollow-fiber or rocking-motion configurations—are already used in GMP manufacturing and can support both suspension and adherent cells under well-controlled shear and oxygen conditions [62] when combined with microcarrier technologies or organoid-based culture, they enable dense cell expansion within compact, sterile environments [63]. Moreover, microfluidic bioreactors offer exceptional control over local nutrient gradients, oxygen tension, and mechanical stimuli—conditions essential for the proliferation and maintenance of sensitive cell types, such as iPSCs or MSCs [64]. Building on their industrial success in efficiency and repeatability, robotics have been applied to automated cellular cultivation: the dual-arm ReBiA system produces *in vitro* tissue models, such as human epidermis, airway epithelium, and intestinal organoids, improving standardization, reproducibility, and cost-effectiveness, and advancing ethical, labor-free research [65]. In addition, Singeç *et al* developed a robotic high-throughput platform for automated human iPSC culture and differentiation under defined conditions, facilitating standardized, scalable production of multiple functional cell types with quality comparable to conventional manual methods [66]. In industry, Multiply Labs (USA) [67] has developed modular robotic systems to automate and scale the manufacturing of cell and gene therapies. These flexible platforms integrate with existing equipment, minimize contamination risks and manual labor, and enable high-throughput production with real-time QC, reducing costs and optimizing cleanroom utilization.

Preserving the functional identity of expanding cells while scaling up remains a central challenge. Existing automated culture and control strategies are not yet capable of adapting to the inherent complexity of living systems, including donor heterogeneity, stochastic cellular behaviors, and dynamic microenvironmental fluctuations. Future self-driving systems should integrate multi-modal, real-time sensing with advanced machine learning to monitor not just morphological parameters but also metabolic activity, differentiation markers, and even transcriptomic or epigenetic states. Feedback-control algorithms should adapt environmental parameters—such as pH, oxygenation, and mechanical cues—in response to cell behavior, ensuring optimal expansion without loss of potency or undesired differentiation. Such platforms will also automate cell quantity and QC and documentation, enabling regulatory-compliant cell manufacturing workflows. These systems could be paired with automated cryopreservation and cell banking units to store batches for future use, allowing full temporal flexibility and consistency in downstream biofabrication.

Ultimately, the vision is a closed-loop, AI-driven cell culture infrastructure that autonomously selects, in-line expands [68], monitors, and delivers specific cell types on demand. This module will form the cellular heart of self-driving bioprinting laboratories, seamlessly integrated with upstream tissue-specific bioprocessing and downstream bioprinting platforms. As advances in robotics, synthetic biology, and computational modeling converge, we anticipate the emergence of intelligent cell foundries capable of rapidly producing billions of clinically relevant cells—tailored to individual patients or applications—thereby transforming the scalability and accessibility of personalized and precision medicine.

### 3.2. Automated bioink synthesis

Building a truly intelligent and autonomous bioink synthesis platform remains one of the most complex and pivotal challenges in biofabrication. Bioinks serve as the foundational interface between engineered constructs and living biology, directly affecting printability, mechanical integrity, and biological performance. Central to the challenge is the inherent multi-dimensionality of bioink design, researchers must navigate a vast design space of biomaterial types, crosslinking chemistries, rheological behaviors, degradation kinetics, and cell–material interactions. These parameters are often highly tissue-specific and nonlinearly coupled, making rational design both time-intensive and empirically driven [69]. Moreover, laboratory-scale bioink production is currently limited to milliliter quantities, far below what is needed for human-scale tissues or organs. Scaling to liter-level or larger volumes is essential, but challenges such as material scarcity, batch variability, and inconsistent properties persist. Integrating automation and AI-assisted technologies could enable high-throughput, standardized bioink synthesis at clinically relevant scales, supporting both commercialization and the clinical translation of biofabricated tissues and organs.

Currently, bioink development often relies on iterative trial-and-error, involving hundreds of formulations to optimize characteristics like shear-thinning behavior for extrusion-based bioprinting, rheopectic behavior for droplet-based bioprinting, viscoelasticity for structural fidelity, and biocompatibility for cell function. To address these limitations, McDonald *et al* have highlighted the potential of applied machine learning as a transformative approach for the rational design of polymeric biomaterials [70]. Rafieyan *et al* proposed one of the most comprehensive open-source datasets on 3D-printed scaffolds, applying over 40 machine learning and deep learning algorithms to predict scaffold quality, printability, and cell responses, thereby enabling more precise and data-driven biofabrication [71]. Moreover, Xu *et al* presented a constraint-based Bayesian optimization framework to predict the viscosity of heterogeneous bioink precursors, enabling efficient, data-driven refinement of extrusion-based bioprinting and reducing the experimental workload required for functional TE [72]. Therefore, the integration of AI and robotics in bioink development—from material selection to process optimization—provides a powerful framework to improve precision, enhance efficiency, and accelerate the fabrication of personalized tissues and organs [73].

Personalized medicine will also rely on on-demand bioinks formulated with a patient’s own cells and ECM-mimetic biomaterials obtained from biopsy samples. Future platforms must be able to integrate Omics data (e.g. proteomics, transcriptomics and metabolomics) to guide the synthesis of personalized bioinks that reflect the native microenvironment of the patient’s tissue. This also opens the opportunity for adaptive material programming, where biomaterials are tuned post-bioprinting via stimuli-responsive elements (light, temperature, pH, enzymes) to mimic developmental processes or pathological remodeling.

In the long term, we envision an intelligent bioink foundry embedded within the self-driving bioprinting laboratories, capable of generating diverse classes of bioinks—hydrogels, ECM-derived materials, synthetic hybrids—in a modular fashion. It will operate as an autonomous formulation factory, where robotic arms, AI-driven experimental design, and digital twins of tissue models converge. Such a system would not only reduce the bottlenecks of current trial-based formulation strategies but also pave the way for evolutionary material design, wherein bioinks have iteratively evolved, tested, validated, documented, monitored and QC with minimal human intervention. This vision aligns with the ultimate goal of precision bioprinting: generating patient-specific, functional tissues and organs on demand.

### 3.3. Intelligent bio-optical and digital reconstruction center

Bioprinting has emerged as a transformative approach for engineering complex biological constructs by precisely organizing living cells (cellular spheroids, organoids, etc.), biomaterials, and bioactive factors into predefined architectures [74]. The choice of bioprinting modality is closely tied to the desired tissue construct and clinical application, as the fidelity, resolution, and bioink requirements vary across platforms [75]. A central challenge remains the accurate translation of digital models into functional biological constructs that can be customized for patient-specific repair of tissue and organ defects, whether external or internal. To enable personalized reconstruction, bioprinting increasingly relies on advanced imaging technologies capable of accurately capturing defect geometry and tissue architecture. Established radiological techniques such as CT and cone-beam CT remain the gold standard for bone and dental applications, while MRI provides superior contrast for soft tissues without radiation exposure [76]. Intraoperative modalities, including O-arm CT, 3D ultrasound, and intraoperative optical coherence tomography (OCT), allow real-time assessment and guidance during surgical procedures [77]. For surface and shallow tissue defects, structured-light and laser scanners, red–green–blue depth (RGB-D) cameras, and emerging smartphone-based light detection and ranging and photogrammetry systems offer rapid, non-invasive acquisition of external anatomy [74]. At the microscale, OCT and photoacoustic imaging provide high-resolution mapping of epithelial layers and vascular features [78], while micro-CT (*µ*CT) is indispensable for preclinical models and *ex vivo* graft assessment [79]. Increasingly, multimodal fusion approaches integrate volumetric scans with surface imaging to create comprehensive, patient-specific models. Together, these technologies provide the essential digital foundation for translating defect morphology into printable constructs, thereby bridging medical imaging with automated bioprinting. Building on this foundation, an intelligent, multifunctional optical and imaging platform—integrated within self-driving bioprinting laboratories—could be deployed in hospitals or dedicated research facilities to acquire high-fidelity data of patient-specific defects and target tissues and organs. Beyond initial data capture, such a center could provide continuous evaluation of bioprinted constructs, enabling real-time feedback for QC, adaptive optimization of bioprinting parameters, and iterative improvement of tissue models. By combining multimodal imaging, automated analysis, and closed-loop feedback, this approach establishes a fully-integrated pipeline that seamlessly connects diagnostic imaging with precision biofabrication.

However, the diversity and structural complexity of human tissues and organs, combined with our still-limited understanding of their multiscale organization, create substantial barriers for accurate digital reconstruction. In addition, resolution and acquisition speed of these imaging technologies are primarily constrained by the inherent properties of the equipment, such as sensor sensitivity, scanning throughput, and penetration depth [80]. As a result, generating high-fidelity 3D models from these datasets remains a significant technical challenge. This process involves complex segmentation, alignment, and surface reconstruction tasks, which must be performed with high precision to ensure the fidelity of the digital model. Moreover, translating these 3D models into executable bioprinting instructions—commonly referred to as G-code—requires careful consideration of the specific bioprinting technology employed. Beyond technical conversion, the design and selection of bioprinting strategies and bioinks depend critically on the target tissue or organ, as well as on the sequence and integration of multiple bioprinting processes. These factors collectively introduce substantial complexity, posing a major challenge for fully automated bioprinting systems. Consequently, an AI-integrated, self-optimizing 3D modeling and control system is critical for self-driving bioprinting laboratories, enabling seamless integration of patient-derived imaging data with the printheads of diverse bioprinting modalities and orchestrating the execution of complex, multi-material fabrication workflows. To empower intelligent bioprinting, advanced AI techniques—including but not limited to machine learning (e.g. decision trees, neural networks), deep learning (e.g. reinforcement learning, convolutional neural networks), expert systems, and natural language processing—are integrated into this system. These technologies enable real-time process optimization, adaptive control, and predictive modeling, thereby enhancing the efficiency and accuracy of bioprinting processes. For example, vascularization and perfusion of bioprinted human-scale tissues and organs remain major bottlenecks, and the ability to accurately map and reconstruct microscale vascular networks within target tissues is limited by the intrinsic resolution and penetration constraints of current imaging and reconstruction technologies. Sexton *et al* reported a model-driven vascular design platform that enables rapid generation of organ-scale vascular trees, integrating multifidelity hemodynamic simulations with digital twins and advanced 3D bioprinting to fabricate perfusable networks that substantially improve viability in large, engineered tissue constructs [81].

### 3.4. Self-driving bioprinting hub

Building upon the foundational advancements above, the next phase in bioprinting is the implementation of intelligent bioprinting processes. These processes are categorized into two primary approaches: *in vitro* and *in vivo* bioprinting, each tailored to specific clinical scenarios and patient needs.

As is well recognized, the use of a single bioink or a sole bioprinting modality is insufficient to fabricate complex, functional tissues *in vitro* [22]. Therefore, achieving intelligent *in vitro* bioprinting requires the integration of multiple advanced bioprinting techniques, crosslinking mechanisms, multimodal sensing platforms, and real-time process monitoring and controlling systems, establishing an intelligent, fully automated, multi-dimensional, and multi-process bioprinting platform that is essential for realizing the vision of self-driving bioprinting. By moving beyond the limitations of conventional 3D bioprinting platforms and simple robotic-arm assistance, the integration of AI-driven, multidimensional, multiscale robotics substantially expands the functional landscape of advanced bioprinting technologies. This convergence marks a pivotal step toward achieving truly multi-cellular, multi-material, multi-process, and multi-dimensional bioprinting. The bioprinting process can be envisioned as an automated production line, analogous to automobile manufacturing, where distinct robotic workstations perform specialized bioprinting tasks in sequence, each contributing to different aspects of the construct’s architecture and function. For instance, the BioAssemblyBot® 400 and 500 (BAB400 and 500, Advanced Solutions Life Sciences, USA) [82] employ a six-axis robotic arm with interchangeable ‘hands’ for dispensing, imaging, environmental control, and more—effectively operating as modular workstations to execute complex bioprinting workflows under autonomous control. Similarly, the Poietis NGB-R^TM^ (France) [83] exemplifies a next-generation robotic bioprinting platform that integrated laser-, droplet- (micro-valve), and extrusion-based bioprinting modalities within a unified architecture incorporating advanced robotics, high-resolution microscopy, and AI-driven feedback control. This platform enables bioprinting with single-cell–level precision, real-time process monitoring, and autonomous parameter adjustment to ensure optimal deposition fidelity. Furthermore, Poietis has developed the NGB-C™, the first clinical-grade bioprinter engineered for deployment in hospital environments under GMP-compliant conditions, representing a significant milestone toward the clinical translation of robotic and automated bioprinting technologies. These innovations exemplify how robotics-driven platforms are pushing bioprinting beyond research laboratories toward clinical translation. Besides, the feasibility and advantages of integrating multi-material, multi-cellular, and multi-process approaches for constructing functional tissue substitutes have already been well-demonstrated in recent studies [84]. As we highlighted in our previous perspective [85], the ‘volumetric bioprinting plus’ (VP^+^ model) concept builds on volumetric bioprinting, which offers rapid fabrication of volumetric structures but is limited in compositional complexity. Integrating it with other bioprinting modalities overcomes these limitations, enabling multi-material, multi-cellular tissue constructs. For example, Ribezzi *et al* combined extrusion-based and volumetric bioprinting to rapidly create complex free-form structures [86], while Größbacher *et al* integrated volumetric bioprinting with melt electrowriting to construct geometrically intricate, multi-material, multi-cellular architectures [87]. A similar strategy could also be applied to acoustics-based bioprinting, further expanding the potential of hybrid approaches for constructing complex, functional tissue architectures. In addition, Zhou *et al* developed an independent multi-nozzle bioprinting platform that integrates extrusion-based and droplet-based bioprinting, and electrospinning techniques to fabricate full-thickness vascularized skin patch models [88]. This platform further expands the biofabrication toolbox by enabling the combination of additional nozzle-based bioprinting modalities for constructing complex tissue architectures. These developments substantiate the feasibility of establishing an intelligent, fully-automated, multi-dimensional, and multi-process bioprinting platform, governed by an AI-integrated, self-optimizing 3D modeling and control system. Such a platform would facilitate patient-specific bioprinting of target tissue defects, incorporating real-time monitoring and QC, feedback, and self-optimization mechanisms to generate functional tissue substitutes. The resulting biofabricated constructs could then be seamlessly transferred via robotic, drones or automated conveyance systems (like magnetic levitation transfer tube) into intelligent bioreactors (section 3.5) for dynamic, biomimetic culture and functional maturation, ultimately preparing them for *in vivo* transplantation.

Conversely, *in vivo* bioprinting enables precise fabrication of tissue constructs that conform to the native anatomical and physiological environment, thereby facilitating site-specific regeneration and repair [89]. *In vivo* bioprinting offers high adaptability, streamlined procedures, and improved cell viability, function, host integration, and aesthetics. By fabricating tissues directly at the target site, it eliminates the need to transport prefabricated constructs, reducing infection and immune rejection risks. At present, as mentioned before, a range of bioprinting approaches have been applied in *in vivo* bioprinting [90], demonstrating their feasibility. Each technique, however, presents distinct advantages and limitations depending on the specific application, target tissue, and bioprinting conditions. For instance, the concept of intracorporeal or endoscopic bioprinting has been developed to enable minimally-invasive intravital tissue fabrication. Using this approach, Urciuolo *et al* demonstrated that cell-laden, photosensitive hydrogel structures could be precisely bioprinted across and within the tissues of live mice, highlighting the potential of *in vivo* bioprinting for direct tissue repair and regeneration [85]. Recently, Davoodi *et al* developed an image-guided deep tissue *in vivo* sound printing platform [91], which uses crosslinking agent–loaded, low-temperature–sensitive liposomes to enable rapid, on-demand crosslinking of diverse biomaterials with focused ultrasound, allowing precise fabrication within deep tissues under real-time imaging guidance.

In addition, specialized bioprinting devices have been developed to translate these modalities into practical *in situ* applications, serving as operational interfaces between bioprinting technologies and the patient’s body. These devices are broadly categorized into handheld bioprinters, frame-based multi-axis systems, and robotic-assisted platforms. For instance, Levin *et al* [92] developed a commercial articulated collaborative *in vivo* bioprinter equipped with a customized printhead and dedicated path-planning software, enabling high-fidelity bioprinting directly onto curved and dynamic body surfaces. Regarding the internal tissues and organs, Thai *et al* reported a multifunctional and flexible *in situ* 3D bioprinter [93], which features a high degree of freedom soft printing head integrated into a flexible robotic arm to deliver multilayered biomaterials to internal organs/tissues. Together, these observations demonstrate that successful *in vivo* bioprinting require careful alignment of the selected bioprinting modality with the anatomical context of the defect, whether in superficial tissues or deep internal organs. Moreover, *in vivo* bioprinting is closely integrated with surgical workflows, serving as a precise and complementary extension of operative procedures for targeted tissue repair and regeneration. Consequently, the integration of bioprinting with advanced surgical robotics to establish a smart, autonomous *in vivo* bioprinting platform—within the framework of self-driving bioprinting laboratories—holds great promise for accelerating clinical translation and broadening therapeutic applications. For instance, the Da Vinci surgical system (Intuitive surgical), a pioneering robotic-assisted platform providing high-definition 3D visualization and unparalleled precision control, offers a compelling foundation for *in situ* bioprinting applications [94]. Widely employed in procedures, such as prostatectomies and cardiac valve repairs, the system’s ergonomic consoles and multi-port minimally invasive access could be leveraged to guide bioink deposition directly onto target tissues. By integrating customized bioprinting printheads and real-time imaging feedback into the Da Vinci workflow, it becomes feasible to perform precise, patient-specific tissue fabrication within the surgical environment, merging the advantages of robotic precision, minimally-invasive access, and automated tissue reconstruction. Additionally, Kim *et al* developed a hierarchical surgical robot transformer (SRT-H) [95] that enables dexterous, long-horizon autonomous surgery using language-conditioned imitation learning. The system successfully executed the first autonomous laparoscopic intestinal anastomosis on a live animal, a complex procedure demanding high precision and real-time adaptation to tissue variations. Integrating bioprinting modules into SRT-H would allow autonomous, real-time deposition of patient-specific tissue constructs, leveraging its AI-driven precision to monitor tissue dynamics and perform suturing, advancing autonomous regenerative surgery.

To execute this smart, autonomous *in vivo* bioprinting platform, a defect is first identified and assessed using the intelligent, multifunctional optical and imaging platform described above (section 3.3), ensuring precise localization and characterization. The imaging data are then uploaded to the AI-integrated, self-optimizing 3D modeling and control system, which generates patient-specific bioprinting instructions. Simultaneously, the recipient is transferred to the *in vivo* bioprinting platform, equipped with supplemental oxygen and vital signs monitoring devices. Upon synchronization between the control system and the autonomous bioprinter, *in vivo* bioprinting is performed with real-time monitoring and feedback, allowing the system to adaptively respond to surgical and bioprinting conditions. Following the procedure, the patient is returned to the clinical bed for post-operative care, with ongoing evaluation and monitoring conducted via the intelligent optical and imaging platform.

### 3.5. Intelligent bioreactor systems

Bioreactors offer a controlled and monitored environment that supports the *ex vivo* engineering of living tissues, allowing for precise regulation of biological and biochemical processes [36, 96]. To move toward intelligent bioreactor systems for autonomous operation, two aspects require special attention. The first involves the maturation or incubation of bioprinted tissue. Simultaneously, the second focuses on creating a closed-loop system capable of providing proper non-destructive monitoring, stimulation, and timely feedback on tissue conditions. Ultimately, the most advanced form of intelligent bioreactors would interconnect multiple patient-derived, iPSC-based tissues, forming a platform that mimics whole-body physiological systems and minimizes immune rejection, thereby enabling highly precise and patient-specific tissue maturation.

Consideration for bioprinted tissue maturation in bioreactors: bioprinted tissues should reach and be maintained at an appropriate level of maturation to be suitable for a subsequent self-driving bioprinting process. For maturation, both physical and biochemical factors need careful consideration and should be specifically tailored to the type of tissue being bioprinted. From a physical consideration, one key factor is the size of the bioreactor. Depending on the transplantation or clinical application, the required dimensions of the bioprinted tissue can vary. Next, circulation and perfusion systems are vital components of bioreactors, crucial for maintaining the viability and maturation of bioprinted tissues [97–102]. In the human body, organs continuously receive sufficient nutrients through blood flow from the heart [103]. Therefore, bioreactors should be equipped with systems that maintain medium flow throughout the entire volume and, when needed, allow direct connection to bioprinted tissues. This functionality can be achieved by integrating an automatic pump system. Some tissues require additional physical stimulation to mature further or achieve stable functional states [101, 102, 104]. For this aspect, bioreactors should be equipped with integrated stimulation modules that provide precise control over the environment. These modules can deliver mechanical, electrical, and sometimes acoustic or ultrasonic stimuli. For instance, to reliably achieve desired tissue features—such as increased ECM formation, stronger mechanical properties, and advanced functional maturity—the bioreactor should operate automatically, with stimulation modules managed by integrated system feedback. When considering biochemical factors in bioreactors, the primary concern is maintaining sterility, as contamination can directly impact tissue integrity and viability. Once sterility is ensured, biochemical factors are largely related to the mass transportation of bioprinted tissues inside a bioreactor. Among these, gas exchange is fundamental in bioreactors, analogous to respiration in living organisms and the regulation of CO_₂_ concentration in conventional cell incubators [96, 105]. For proper tissue maturation, gases must be carefully managed through integrated gas sensors within the bioreactor. In addition to gases, tissues consume a wide range of nutrients, including amino acids, proteins, and supplements, which must be supplied in an appropriately formulated culture medium [101, 106]. Because nutrient demands differ according to tissue type and metabolic requirements, bioreactors should provide tailored medium compositions.

In the future, intelligent bioreactors designed for specific tissue types should evolve toward integrated systems. This evolution of bioreactors, progressing from isolated units to interconnected platforms, can be conceptualized in stepwise levels (table S1. Supplementary). At *Level 0*, an individual bioreactor is dedicated to a single tissue type. Although these bioreactors enable tissue maturation sufficient for transplantation, they remain limited in their capacity to replicate the systemic interactions that occur within the human body, where organs are interconnected and function through complex interrelationships. To provide similar physiological tissue conditions, future systems should enable communication between individual bioreactors. By interconnecting bioreactors and allowing bioprinted tissues to undergo incubation and maturation within these coupled platforms, the resulting outputs would more closely reflect patient-specific physiological conditions. This concept parallels the integration of multiple organ-on-a-chip platforms, which have demonstrated enhanced capabilities for investigating inter-organ interactions and systemic effects [103, 107, 108]. To envision the higher levels of bioreactor design, imagine a system that incorporates seven distinct bioreactors corresponding to the brain, heart, lung, liver, kidney, intestine, and bone. These bioreactors are interconnected through pipelines and circulation modules that reproduce physiological relationships observed in the human body. In this framework, patient-derived bioprinted heart tissue is incubated and matured in a heart-specific bioreactor for transplantation. The *Level 1* intelligent bioreactor system represents an integrated platform in which all bioreactors are connected and designed to replicate only the physical properties of tissues rather than containing complete biological constructs. Each bioreactor, apart from the one that is designated for the bioprinted tissue of interest, provides auxiliary physiological functions, such as dialysis in a kidney-specific bioreactor. Biochemical parameters within the *Level 1* system are regulated within a defined baseline range, and the circulating culture medium flows across all bioreactors in a manner analogous to blood circulation. The circulating medium contains diverse growth factors at concentrations tailored to the conditions of each bioreactor, and condition data obtained from the closed-loop monitoring system of the heart bioreactor are used to adjust the operational parameters of all other bioreactors, thereby ensuring coordinated and physiologically relevant maturation of the tissue.

Beyond *Level 1*, the *Level 2* system incorporates basic tissues, represented by specific cell-based bioprinted constructs, within each bioreactor. While maintaining the operational framework of the *Level 1* system, the *Level 2* system introduces biological aspects by enabling cellular activity within constructs. Tissue function and inter-organ communication arise from the biochemical products secreted by the cells that constitute each tissue. Although the *Level 2* system provides additional biological fidelity, it may not fully replicate patient-specific conditions because most of the incorporated tissues are not derived from patient cells, which can trigger undesired immune responses. Nevertheless, constructs maintained in the *Level 2* system consume nutrients from the circulating medium and generate biochemical products that influence the function of other tissues within the interconnected bioreactors. At this level, the feedback systems are required to monitor the reference tissue (non-patient-specific tissue) in each bioreactor and adjust operational parameters based on the recorded conditions. The ultimate concept for intelligent bioreactors in self-driving bioprinting is represented by the *Level 3* system, in which all bioreactors contain patient-derived or iPSC-based bioprinted tissues. Even if a patient requires only a single type of tissue for transplantation, the functionality of the construct generated in a *Level 2* system does not match that of the native tissue, and the Level 3 system remains insufficient to reproduce the original tissue properties in full. However, by progressively integrating additional bioreactors and refining their interconnections, the *Level 3* system has the potential to approximate native tissue functions with high precision while avoiding adverse immune responses.

Closed-loop feedback and non-destructive monitoring of bioprinted tissue condition in bioreactors: bioreactors will require further integration to monitor the condition of bioprinted tissues [106, 109]. In addition, the condition of bioprinted tissues must be non-destructively evaluated at multiple biological levels to ensure successful transplantation or clinical application, including gene expression, protein and cellular states, minimal functional units, and overall tissue performance. To enable such closed-loop feedback and monitoring, bioreactors should incorporate and automatically perform diverse analytical techniques capable of continuously tracking these parameters.

In bioprinted tissues, gene-level signatures are the earliest and most sensitive indicators of maturation and lineage specification [110, 111]. In the case of bioprinted tissues derived from stem cells, transcriptional fidelity must be validated to ensure that cells differentiate into the intended lineages. Real-time gene expression monitoring can be performed on DNA or RNA obtained through a biopsy channel using reverse transcription quantitative polymerase chain reaction or RNA sequencing [112]. Meanwhile, emerging non-invasive molecular sensors, such as Raman-based approaches, can provide timely feedback on tissue quality [113, 114]. These signatures not only validate the biological direction of bioprinted tissues but also help detect off-target differentiation, stress responses, incomplete maturation, or canceration. Protein-level analysis provides a crucial bridge between transcriptional activity and the functional architecture of bioprinted tissues. Samples acquired through a biopsy channel can be analyzed using advanced proteomic techniques [115–117]. Complementary non-invasive approaches, such as Raman or infrared spectroscopy and advanced imaging modalities, allow protein-level monitoring without destructive sampling, providing real-time insights into protein conformation, localization, and dynamics in native contexts [118, 119]. The cellular-level monitoring ensures viability, stability, and translational potential as tissues face mechanical stress, nutrient imbalance, and interactions with novel biomaterials [120]. Key parameters include cellular morphology, proliferation, spatial organization of multiple cell types, viability, and metabolic activity. Similar to different levels, the assessment of these parameters can be categorized into biopsy-based analyses and non-invasive approaches. Through the biopsy channel, assays provide quantitative insights into survival, growth, differentiation, and long-term stability [121–124]. In contrast, non-invasive approaches, including live-cell imaging of morphology, label-free assessment of multicellular spatial distribution, and flux-based metabolic monitoring sensors, enable dynamic, real-time evaluation without disturbing structural integrity [125–129]. By combining biopsy-derived depth with non-invasive continuity, bioreactors can establish closed-loop feedback systems that adaptively regulate culture conditions, thereby preserving the intended biological function of bioprinted tissues throughout maturation.

Bioprinted tissues advance from early cellular phenomena to the emergence of localized functional domains and ultimately to coordinated tissue/organ-level functions. Within this tissue/organ frame, these domains are the smallest coherent assemblies that carry out specific functions and serve as practical checkpoints for maturation. Since biopsy-based analyses were addressed at earlier levels, monitoring at the tissue/organ-level should prioritize non-invasive approaches during the final preclinical stage preceding clinical application, including transplantation. These approaches can be grouped into two categories, namely non-invasive imaging modalities and sensor-based systems that can be integrated into a bioreactor without disrupting structural or functional integrity. Non-invasive imaging modalities that preserve structural integrity, such as OCT and ultrasound, can complement these readouts [130, 131]. Monitoring at this scale benefits from sensors that are already compatible with clinical use and can be easily incorporated into bioreactors, including oxygen and pH optodes, electrochemical metabolite probes, microelectrode arrays for electrophysiology, and strain or force transducers for mechanical measurements [128, 129, 132, 133] miniaturized versions of these sensors are increasingly applied to organoids, enabling measurements at the scale of mini-organs without disrupting their architecture [134]. Transient (or biodegradable) devices offer an additional option for monitoring tissues/organs in bioreactors. Such devices operate for a defined period, capture targeted signals, and subsequently dissolve without leaving harmful residues [134–136]. Because these sensors can be embedded within bioprinted tissues with minimal perturbation, feedback control during maturation is supported. The devices can remain associated with the graft during the transition to clinical use, which extends monitoring into the early post-implant phase. This strategy reduces the need for secondary procedures to remove hardware and maintains continuity between preclinical conditioning and clinical assessment. In turn, feedback from these measurements should guide targeted adjustments to physical/biochemical parameters within the bioreactor, thereby accelerating maturation. At the same time, selected interfaces may also deliver controlled inputs directly to bioprinted tissues, coupling measurement with intervention and strengthening readiness for clinical use.

The bioreactors govern the incubation and maturation of bioprinted tissues, maintaining their quality and stability until clinical application through precise regulation of physical and biochemical factors. Environmental variables must be automatically controlled in a closed-loop manner. At the same time, the condition of bioprinted tissues themselves should be monitored with high granularity and provided with multiscale monitoring, QC and feedback, spanning from gene and protein signatures to cellular organization and tissue-level function. Such feedback must be coordinated with environmental control for unified management, creating a seamless loop between sensing and actuation. The progressive development of these intelligent systems will generate vast datasets required for monitoring and maintaining tissue maturation across interconnected platforms. To address this complexity, AI integration will be indispensable, enabling rapid decision-making, predictive modeling, and rigorous data management for adaptive control. Ultimately, these systems could establish a comprehensive tissue information library, serving as a reference framework to rapidly adjust bioreactor conditions for new patients and their individualized therapeutic needs.

### 3.6. Self-driving transplantation and clinical applications

Once tissues and organs are matured within bioreactors, the subsequent stage involves their safe and precise transplantation into patients. AI-enhanced robotic surgery provides the necessary accuracy and control for such delicate procedures. These technologies can lower surgical difficulties, minimize tissue damage, and speed up patient recovery. A major step toward fully autonomous, patient-specific transplantation is creating a seamless process, from organ design and growth to clinical implantation, by adding an intelligent, automatic robotic transplantation center as another important part of self-driving bioprinting laboratories. Robotic technology and AI have revolutionized surgery by improving accuracy, productivity, and patient outcomes. In transplant surgery, AI supports donor-recipient matching, real-time surgical decision-making, and post-operative care, while robotic systems enhance mobility, vision, and tremor-free accuracy. Many robotic systems have been used for surgical operations. Systems like the da Vinci Surgical System have already shown success in kidney and liver transplants, reducing complications, blood loss, and recovery times. Robotic-assisted kidney transplantation (RAKT) offers a minimally invasive alternative to open surgery, though evidence on long-term outcomes has been limited. In 624 living-donor recipients (2015–2023), RAKT demonstrated low intraoperative complication rates (1.1%) and favorable perioperative safety. High-grade early and late post-operative complications occurred in 7.7% and 2.3% of patients, respectively, with graft nephrectomy required in 1.9%. Long-term follow-up showed low rates of dialysis and mortality, with no deaths attributed to RAKT [137]. These findings highlight the safety, efficacy, and functional durability of RAKT, supporting its broader application in kidney transplantation. Recent progress in surgical robotics highlights the growing potential of integrating automated transplantation into the bioprinting pipeline. For instance, Medtronic’s Hugo™ robotic-assisted surgery system has recently been evaluated in large multicenter clinical trials for urologic procedures, showing safety and effectiveness comparable to established robotic platforms [138].

After implantation, the most critical step is long-term monitoring of the function of implanted organs within host body, as timely detection of rejection determines graft survival and patient outcomes. Currently, monitoring relies heavily on invasive biopsies, such as endomyocardial or renal biopsies, which remain the clinical gold standard for detecting rejections [139, 140]. While effective in providing histological confirmation, these procedures are associated with several limitations, including risks of bleeding, infection, and vascular injury, as well as sampling errors due to tissue heterogeneity. In addition, repeated biopsies impose significant discomfort and psychological stress on patients, increase healthcare costs, and may delay timely detection of early rejection events [141, 142]. These challenges highlight the urgent need for less invasive, continuous, and patient-friendly monitoring strategies. In response, several non-invasive alternatives are under investigation, including advanced imaging modalities, circulating, and emerging biosensor technologies. Among these, biosensor-integrated platforms capable of real-time physiological monitoring, such as flexible patches using electrical impedance spectroscopy, enable non-invasive and continuous detection of tissue-level changes associated with rejection. Designed with advanced electrode materials and conformable architectures, these technologies hold promise for early diagnosis, reduced biopsy dependence, and improved long-term transplant outcomes [143, 144]. Building on this, integration of AI-driven analysis allows multimodal data including sensor outputs and imaging from MRI, CT, or ultrasound to be computationally evaluated for functional insights. Such approaches can correlate physiological signals with structural integrity, blood vessel development, and cellular distribution, creating a comprehensive and predictive monitoring framework for engineered and transplanted tissues.

These techniques detect early indications of abnormal development, damage, or tissue death. A real-time health assessment for the implanted tissue is produced by combining data from implanted biosensors that detect physiological factors like pH, glucose, metabolites, and oxygen levels. Predictive models anticipate potential complications, and thus adjust external support mechanisms, such as perfusion devices or localized drug delivery, based on tissue requirements. Further, intraoperative assessment via AI provides real-time QC and feedback on tissue handling, positioning, and vascular integration, reducing errors and optimizing surgical outcome [145]. Furthermore, AI-guided simulation and feedback systems enable rehearsal of transplantation procedures using patient-specific organ models, improving skill acquisition and procedural reproducibility.

Projecting the timeline and major milestones to clinical translation: bioprinting has advanced rapidly, where complex tissue structures, including ears, muscles, and vascularized constructs, have been demonstrated, bringing the field closer to clinical translation [146–148]. Today, many bioprinted tissues remain in preclinical or early clinical stages, with bioartificial organs, bioprinted skin, and vascularized tissues approaching human applications [149]. The growing 3D bioprinting market reflects increasing momentum, though challenges persist, including vascularization, cell survival, scalable manufacturing, biomaterial innovation, and regulatory approval. A recent milestone toward clinical translation is the development of the LIGŌ 3D bioprinting device, which has successfully entered clinical trials in Australia. Unlike conventional skin grafting, LIGŌ prints patient-derived cells in the form of ‘skin-growing ink’ directly onto wounds, enabling highly precise, layer-by-layer regeneration of damaged tissue. The system functions much like an inkjet printer but dispenses living bioinks tailored to the geometry and depth of each defect, guided by real-time imaging. Early clinical outcomes suggest accelerated wound closure, reduced complications, and shorter hospital stays, highlighting the therapeutic potential of such point-of-care bioprinting systems. Importantly, LIGŌ also exemplifies the broader trajectory toward self-driving transplantation platforms, where autonomous, GMP-compliant devices are deployed at the bedside to deliver personalized, regenerative therapies with minimal manual intervention [150]. Furthermore, self-driving bioprinting platforms are expected to accelerate these developments by integrating AI-driven design, real-time monitoring, and adaptive bioprinting [151]. In the near term, less complicated tissues such as cartilage and vascularized patches for drug testing are expected to achieve regulatory approval and enter clinical practice [152]. In the mid-term, more complex tissues may be implanted in humans. Over the long term, functional organs such as kidneys, livers, lungs, and hearts with vascular networks and metabolic functions could provide personalized alternatives to donor transplants, reducing organ shortages. Advanced self-driving laboratories may produce patient-specific grafts integrated with robotic surgical systems for precise transplantation, potentially transforming organ replacement and redefining standards of care [153, 154].

## 4. Ethical and societal concerns of self-driving bioprinting laboratories

Ethical considerations for self-driving bioprinting laboratories are critical throughout the whole process, including pre-printing: cellular types and biomaterials, bioprinting process, and post-bioprinting to clinical applications. Broadly, these considerations fall into two main categories: those related to the bioprinting process itself and those associated with the use of AI technologies. Several strategies aligned with the principles of *Ethics-by-Design, transparency, oversight, regulation, and public engagement* are critical.

The goal of ethics-by-design is to specifically incorporate ethical consideration and assessment into each phase of the development of bioprinting and AI. The bioprinting process raises ethical concerns primarily related to the sourcing of bioinks, including those derived from animals, humans, plants, marine organisms, bacteria, and other microorganisms. These raise questions of human dignity and identity, informed consent, and the broader implications of sourcing biological material responsibly. Similar to RM, ethical challenges arise in relation to animal experimentation, sourcing of cells, and biobanking. It is crucial to provide informed consent and handle genetic and medical data securely. Strong monitoring and well-defined ownership requirements help guarantee that materials are utilized only for valid scientific objectives [155–158].

In parallel, AI integrated into self-driving laboratories must be designed with strict safeguards that prevent unsafe or unethical experimentation. Ensuring that machine autonomy never compromises patient safety, scientific validity, or societal trust is essential to responsible innovation.

Transparent consent processes: these are essential to maintain trust and protect donors and patients. Biofabrication relies on human-derived samples and sensitive data, including genetic profiles and medical histories. Donors must be informed of the potential uses of their materials, including AI-driven analyses, commercial applications, and future clinical translation. In clinical trials, patients must understand both the immediate and long-term risks, including unknown effects of regenerative therapies, tumorigenicity, immune reactions, or complications from bioinks [159, 160].

Independent oversight: AI is driving innovation across clinical research and healthcare, yet its rapid utilization introduces risks of bias, opacity, and ethical uncertainty. Independent oversight is critical to ensuring these technologies are used responsibly and remain aligned with human values. Oversight committees, composed of ethicists, patient advocates, clinicians, and AI experts, provide a multidisciplinary safeguard that internal reviews alone cannot guarantee. Their role is to evaluate the fairness of algorithms, protect participant autonomy, and ensure that consent processes reflect the unique risks of AI-enabled studies. Recent initiatives, such as the Framework for Review of Clinical Research Involving AI by the multi-regional clinical trials Center and WIRB-Copernicus Group, highlight how structured oversight can adapt to challenges like algorithmic bias, adaptive learning, and data privacy. By applying stage-specific review criteria and emphasizing human-in-the-loop mechanisms, such committees ensure that AI complements rather than replaces human judgment. Ultimately, independent oversight not only strengthens accountability and transparency but also builds public trust in AI-driven research and applications [161–163].

Regulatory innovation is essential to manage the intersection of AI and bioprinting, where traditional frameworks are often inadequate. New guidelines must define safety standards for automated experimentation, specify acceptable levels of AI autonomy, and ensure reproducibility and long-term monitoring. These measures are critical for clinical translation, supporting ethical trial design, equitable patient selection, and fair access to emerging therapies. Early-stage clinical studies must advance innovation without compromising patient safety or public trust. Data quality and fairness remain major concerns. AI systems depend on training datasets that are frequently incomplete, biased, or skewed toward successful outcomes. Sensor or equipment errors can further distort data, causing cascading algorithmic failures. Mitigation requires strong validation pipelines, cross-verification, and testing across diverse experimental conditions [164, 165]. Self-driving laboratories also raise questions of accountability. When AI-driven systems make errors whether in therapeutic decisions, experimental outputs, or resource use, it is unclear who bears responsibility. This highlights the need for governance frameworks that clearly define liability in AI-enabled science. Experimental safety is another priority. Small deviations in automated reagent handling or environmental control can compromise results. Finally, strong data security and privacy protections are imperative. Biofabrication frequently involves sensitive, patient-specific information that is vulnerable to misuse. Robust data governance, regulatory compliance, and cybersecurity investment are essential to protect privacy and maintain public trust in AI-driven research [160, 166].

Public engagement: It is crucial to ensure that societal values influence research priorities and ethical boundaries in AI and biofabrication. These technologies raise profound questions about human augmentation, the blurred line between therapy and enhancement, and the possibility that the public will misinterpret scientific findings. For instance, such systems could fabricate any human organ with precision, and in the far future, might even be capable of producing entire human. This could challenge fundamental concepts of human identity, life, and longevity. Meaningful public engagement through education, consultation, and transparency allows communities to voice concerns, shape decisions, and build trust. Evidence shows such participation improves accountability and prevents harm by integrating diverse perspectives. In AI-driven laboratories, it guides responsible innovation and ensures progress aligns with societal values [167–169].

Ethical management in AI-driven biofabrication requires a proactive approach that balances innovation with societal responsibility. Through ethics-by-design, transparent consent, independent oversight, regulatory innovation, and public engagement, researchers can advance technology while protecting dignity, ensuring safety, and maintaining public trust.

## 5. Concluding insights and frontier challenges in self-driving bioprinting

Motivated by the growing expectation that advanced bioprinting technologies will overcome critical limitations in TE and RM, this perspective highlights the transformative potential of self-driving bioprinting laboratories for next-generation healthcare. We have proposed an ambitious yet feasible framework for self-driving bioprinting laboratories, integrating critical subsystems—including autonomous cell culture, adaptive bioink formulation, intelligent optical and digital reconstruction platforms, AI-guided bioprinting, intelligent bioreactors, and robotic transplantation—and discussed how their seamless integration within a closed-loop ecosystem could harmonize complex workflows, enhance reproducibility, and accelerate the clinical translation of engineered tissues and organs. Furthermore, we emphasized the critical need for comprehensive ethical, regulatory, and data governance frameworks, along with supportive legislation, to ensure safety, transparency, and equitable access in the deployment of such intelligent bioprinting laboratories. Looking ahead, we envision self-driving bioprinting laboratories as continuously self-learning and adaptive systems capable of standardizing patient-specific tissue biofabrication, minimizing costs and resource utilization, and fundamentally transforming the development and delivery of regenerative therapies.

Facing the reality of this ambitious vision, several critical challenges remain. Technically, self-driving bioprinting laboratories integrate highly heterogeneous subsystems—including automated cell culture, bioink synthesis, intelligent modeling platforms, AI-guided bioprinting, smart bioreactors, and robotic transplantation—necessitating seamless physical integration, precise coordinated control, multimodal data fusion, and real-time sensing with minimal latency and noise. Ensuring consistent batch-to-batch reproducibility, robust hardware synchronization, and comprehensive end-to-end standardization of data formats, communication protocols, and quality metrics remains a major obstacle. Biologically, incomplete understanding of the multiscale complexity of human tissues—including cellular organization, ECM composition, and organ-level architecture—limits faithful recapitulation of tissue function. Knowledge gaps in tissue and organ development, growth mechanisms, and dynamic cellular interactions and uncertainty, coupled with insufficient high-resolution characterization strategies at subcellular and microscale levels, constrain the replication of tissue heterogeneity. Fundamental barriers such as vascularization, innervation, immune compatibility, and the integration of nanoscale extracellular cues with macroscale organ structures must be systematically addressed to achieve functional, clinically relevant constructs. AI and data-driven approaches are indispensable to self-driving bioprinting laboratories but remain constrained by the scarcity of robust, validated models, limited high-quality datasets (e.g. bioink formulations and tissue-specific parameters), high annotation costs, limited model interpretability, and incomplete digital twin representations. These limitations necessitate iterative human-in-the-loop oversight during early deployment, highlighting the importance of progressive model refinement and integration with experimental feedback to achieve reliable, autonomous operation. Regulatory, evaluation, ethical, and safety considerations—including alignment with GMP standards, standardized evaluation metrics and outcome indicators for a self-driving bioprinting system, algorithmic accountability, traceability of source materials, autonomous monitoring, and robust patient-data governance—are critical to maintain public trust and ensure clinical safety. Building self-driving bioprinting laboratories also introduces substantial translational and economic barriers, as establishing such autonomous systems requires significant financial investment, prolonged development timelines, and highly specialized expertise spanning robotics, AI, bioink, bioprinting, and regulatory science. These requirements make implementation challenging for individual laboratories or institutions. A feasible path forward will likely rely on coordinated efforts among major pharmaceutical companies, biotechnology start-ups, and academic–industry consortia, supported by national research foundations and healthcare innovation programs, to pool resources, infrastructure, and multidisciplinary talents needed to realize such complex systems. Importantly, rigorous assessment of the real-world value of self-driving bioprinting laboratories is necessary to balance substantial investments against expected outcomes, commercialization potential, societal benefits, and alignment with national healthcare strategies and future medicine priorities.

To accelerate progress toward this vision, research efforts should focus on high-fidelity tissue modeling, enhancing the precision and scalability in high-throughput bioprinting, real-time adaptive control, AI-assisted development and optimization of bioinks and cellular processes, establishment of comprehensive biomaterialomics databases, digital transformation of experimental data, standardization and interoperability, and strategies to address vascularization, innervation, and immune integration challenges. Concurrently, robust evaluation metrics, ethical frameworks, and sustainable economic models must be established. Tackling these multifaceted challenges through coordinated, interdisciplinary approaches is essential to realize the potential of self-driving bioprinting laboratories, enabling reproducible, scalable, and patient-specific TE and accelerating the clinical translation of regenerative therapies.

## Data Availability

All data that support the findings of this study are included within the article (and any supplementary files). Supplementary Data 1 available at https://10.1088/1758–5090/ae3645/data1.

## References

[1] Atala A, Kurtis Kasper F, Mikos A G (2012). Engineering complex tissues. Sci. Transl. Med..

[2] Loewa A, Feng J J, Hedtrich S (2023). Human disease models in drug development. Nat. Rev. Bioeng..

[3] Hoang V T (2025). Tissue engineering and regenerative medicine: perspectives and challenges. MedComm.

[4] Murphy S V, De Coppi P, Atala A (2020). Opportunities and challenges of translational 3D bioprinting. Nat. Biomed. Eng..

[5] Sun W (2020). The bioprinting roadmap. Biofabrication.

[6] Klebe R J (1988). Cytoscribing: a method for micropositioning cells and the construction of two- and three-dimensional synthetic tissues. Exp. Cell Res..

[7] Hsieh H (2004). Ultra-high-throughput microarray generation and liquid dispensing using multiple disposable piezoelectric ejectors. SLAS Discovery.

[8] Zhang P, Abate A R (2020). High-definition single-cell printing: cell-by-cell fabrication of biological structures. Adv. Mater..

[9] Guo F (2016). Three-dimensional manipulation of single cells using surface acoustic waves. Proc. Natl Acad. Sci. USA.

[10] Zhang Y S, Haghiashtiani G, Hübscher T, Kelly D J, Lee J M, Lutolf M, McAlpine M C, Yeong W Y, Zenobi-Wong M, Malda J (2021). 3D extrusion bioprinting. Nat. Rev. Method Primers.

[11] Gupta D, Derman I D, Xu C, Huang Y, Ozbolat I T (2025). Droplet-based bioprinting. Nat. Rev. Method Primers.

[12] Chichkov B (2022). Laser printing: trends and perspectives. Appl. Phys. A.

[13] Levato R, Dudaryeva O, Garciamendez-Mijares C E, Kirkpatrick B E, Rizzo R, Schimelman J, Anseth K S, Chen S, Zenobi-Wong M, Zhang Y S (2023). Light-based vat-polymerization bioprinting. Nat. Rev. Method Primers.

[14] He C F, Qiao T H, Wang G H, Sun Y, He Y (2025). High-resolution projection-based 3D bioprinting. Nat. Re. Bioeng..

[15] Kopyeva I, Brady R P, DeForest C A (2025). Light-based fabrication and 4D customization of hydrogel biomaterials. Nat. Re. Bioeng..

[16] Garciamendez-Mijares C E, Ruiz D S R, Kuang X, Halabe A S G, Gonzalez B S, Ruiz C G R, Mestre F L, Nunes F C, Zhang Y S (2025). Acoustic bioprinting: a glimpse into an emerging field. Small Methods.

[17] Melde K, Athanassiadis A G, Missirlis D, Shi M, Seneca S, Fischer P (2024). Ultrasound-assisted tissue engineering. Nat. Re. Bioeng..

[18] Groll J (2018). A definition of bioinks and their distinction from biomaterial inks. Biofabrication.

[19] Zhang Y S, Khademhosseini A (2017). Advances in engineering hydrogels. Science.

[20] Decante G, Costa J B, Silva-Correia J, Collins M N, Reis R L, Oliveira J M (2021). Engineering bioinks for 3D bioprinting. Biofabrication.

[21] Schwab A, Levato R, D’Este M, Piluso S, Eglin D, Malda J (2020). Printability and shape fidelity of bioinks in 3D bioprinting. Chem. Rev..

[22] Hospodiuk M, Dey M, Sosnoski D, Ozbolat I T (2017). The bioink: a comprehensive review on bioprintable materials. Biotechnol. Adv..

[23] Qin Y (2024). Biofabrication: bioprinting process, printing materials, and the frontier applications in biomedicine. Addit. Manuf. Front..

[24] Liu A P (2022). The living interface between synthetic biology and biomaterial design. Nat. Mater..

[25] Zhang Z, Zhou X, Fang Y, Xiong Z, Zhang T (2025). AI-driven 3D bioprinting for regenerative medicine: from bench to bedside. Bioact. Mater..

[26] Mota C, Camarero-Espinosa S, Baker M B, Wieringa P, Moroni L (2020). Bioprinting: from tissue and organ development to *in vitro* models. Chem. Rev..

[27] Tanna J, McCann C D, Smith R, Pitino A, Asgedom A, Kong S L, Weiner Y L, Bushnell K, Webb J, Hanley P J (2024). Environmental monitoring of current good manufacturing practices cleanroom facilities for manufacturing of cellular therapy products in an academic hospital setting. Cytotherapy.

[28] Aijaz A (2018). Biomanufacturing for clinically advanced cell therapies. Nat. Biomed. Eng..

[29] Stevens K R (2017). *In situ* expansion of engineered human liver tissue in a mouse model of chronic liver disease. Sci. Transl. Med..

[30] Ogoke O, Oluwole J, Parashurama N (2017). Bioengineering considerations in liver regenerative medicine. J. Biol. Eng..

[31] Jones R C (2022). The tabula sapiens: a multiple-organ, single-cell transcriptomic atlas of humans. Science.

[32] He S (2020). Single-cell transcriptome profiling of an adult human cell atlas of 15 major organs. Genome Biol..

[33] Skardal A (2015). Bioprinting essentials of cell and protein viability. Essentials of 3D Biofabrication and Translation.

[34] Abbott R D, Kaplan D L (2015). Strategies for improving the physiological relevance of human engineered tissues. Trends Biotechnol..

[35] Jiang W, Wu Z, Gao Z, Wan M, Zhou M, Mao C, Shen J (2022). Artificial cells: past, present and future. ACS Nano.

[36] Sarkar N, Bhumiratana S, Geris L, Papantoniou I, Grayson W L (2023). Bioreactors for engineering patient-specific tissue grafts. Nat. Rev. Bioeng..

[37] Parrish J, Lim K, Zhang B, Radisic M, Woodfield T B F (2019). New frontiers for biofabrication and bioreactor design in microphysiological system development. Trends Biotechnol..

[38] Chen D X B (2025). Biomaterials and bioinks for bioprinting. Extrusion Bioprinting of Scaffolds for Tissue Engineering.

[39] Moroni L, Burdick J A, Highley C, Lee S J, Morimoto Y, Takeuchi S, Yoo J J (2018). Biofabrication strategies for 3D *in vitro* models and regenerative medicine. Nat. Rev. Mater..

[40] Gu Y, Forget A, Shastri V P (2022). Biobridge: an outlook on translational bioinks for 3D bioprinting. Adv. Sci..

[41] Ozbolat I T, Yu Y (2013). Bioprinting toward organ fabrication: challenges and future trends. IEEE Trans. Biomed. Eng..

[42] Fletcher J, Miskovic D (2021). Digital and 3D printed models for surgical planning. Digital Surgery.

[43] von Witzleben M (2025). Additive manufacturing of patient-specific, biphasic implants with zonal design for regeneration of osteochondral defects–critical evaluation of the work flow from clinical MRI data to implantation. Mater. Today Bio.

[44] Cui H, Nowicki M, Fisher J P, Zhang L G (2017). 3D bioprinting for organ regeneration. Adv Healthcare Mater..

[45] Singh S, Choudhury D, Yu F, Mironov V, Naing M W (2020). *In situ* bioprinting—bioprinting from benchside to bedside?. Acta Biomater..

[46] Zhang Y S, Dolatshahi-Pirouz A, Orive G (2024). Regenerative cell therapy with 3D bioprinting. Science.

[47] Gantenbein B, Illien-Jünger S, Chan S C, Walser J, Haglund L, Ferguson S J, Iatridis J C, Grad S (2015). Organ culture bioreactors—platforms to study human intervertebral disc degeneration and regenerative therapy. Curr. Stem Cell Res. Ther..

[48] Lim D (2022). Bioreactor design and validation for manufacturing strategies in tissue engineering. Biodes. Manuf..

[49] Bijonowski B M, Miller W M, Wertheim J A (2013). Bioreactor design for perfusion-based, highly vascularized organ regeneration. Curr. Opin. Chem. Eng..

[50] Meneses J, Fernandes S R, Silva J C, Ferreira F C, Alves N, Pascoal-Faria P (2023). JANUS: an open-source 3D printable perfusion bioreactor and numerical model-based design strategy for tissue engineering. Front Bioeng Biotechnol.

[51] Yeo M, Sarkar A, Singh Y P, Derman I D, Datta P, Ozbolat I T (2023). Synergistic coupling between 3D bioprinting and vascularization strategies. Biofabrication.

[52] Pagan E (2023). A handheld bioprinter for multi-material printing of complex constructs. Biofabrication.

[53] Weber M, Park K B, Afshar S (2025). Equalizing access: how robotics and AI can transform surgical care worldwide. Sci. Robot..

[54] Petrus-Reurer S, Romano M, Howlett S, Jones J L, Lombardi G, Saeb-Parsy K (2021). Immunological considerations and challenges for regenerative cellular therapies. Commun. Biol..

[55] Mirshafiei M, Rashedi H, Yazdian F, Rahdar A, Baino F (2024). Advancements in tissue and organ 3D bioprinting: current techniques, applications, and future perspectives. Mater. Des..

[56] Montano M (2014). Translational Biology in Medicine.

[57] Cerneckis J, Cai H, Shi Y (2024). Induced pluripotent stem cells (iPSCs): molecular mechanisms of induction and applications. Signal Transduct. Target Ther..

[58] Xu J, Du Y, Deng H (2015). Direct lineage reprogramming: strategies, mechanisms, and applications. Cell Stem Cell.

[59] Bertaux F, Sosa-Carrillo S, Gross V, Fraisse A, Aditya C, Furstenheim M, Batt G (2022). Enhancing bioreactor arrays for automated measurements and reactive control with ReacSight. Nat. Commun..

[60] Gelinsky M, Bernhardt A, Milan F (2015). Bioreactors in tissue engineering: advances in stem cell culture and three-dimensional tissue constructs. Eng. Life Sci..

[61] Anon CellXpress.ai automated cell culture system| molecular devices. https://www.moleculardevices.com/products/3d-biology/cellxpress-ai-automated-cell-culture-system.

[62] Ganeeva I, Zmievskaya E, Valiullina A, Kudriaeva A, Miftakhova R, Rybalov A, Bulatov E (2022). Recent advances in the development of bioreactors for manufacturing of adoptive cell immunotherapies. Bioengineering.

[63] Chen X Y, Chen J Y, Tong X M, Mei J G, Chen Y F, Mou X Z (2020). Recent advances in the use of microcarriers for cell cultures and their ex vivo and *in vivo* applications. Biotechnol. Lett..

[64] Chen H, Fang Y, Luo Z, Wang Y, Sun W, Zhao Y (2025). Emerging microfluidics for bioreactor. Mater. Today.

[65] Königer L (2024). ReBiA—robotic enabled biological automation: 3D epithelial tissue production. Adv. Sci..

[66] Tristan C A (2021). Robotic high-throughput biomanufacturing and functional differentiation of human pluripotent stem cells. Stem Cell Rep..

[67] Anon Multiply labs—robots making precision medicine. https://multiplylabs.com/.

[68] Fung Shek C, Betenbaugh M (2021). Taking the pulse of bioprocesses: at-line and in-line monitoring of mammalian cell cultures. Curr. Opin. Biotechnol..

[69] Mathur V, Agarwal P, Kasturi M, Srinivasan V, Seetharam R N, Vasanthan K S (2025). Innovative bioinks for 3D bioprinting: exploring technological potential and regulatory challenges. J. Tissue Eng..

[70] McDonald S M, Augustine E K, Lanners Q, Rudin C, Catherine Brinson L, Becker M L (2023). Applied machine learning as a driver for polymeric biomaterials design. Nat. Commun..

[71] Rafieyan S, Ansari E, Vasheghani-Farahani E (2024). A practical machine learning approach for predicting the quality of 3D (bio)printed scaffolds. Biofabrication.

[72] Xu Y, Sarah R, Habib A, Liu Y, Khoda B (2024). Constraint based Bayesian optimization of bioink precursor: a machine learning framework. Biofabrication.

[73] Chen H, Zhang B, Huang J (2024). Recent advances and applications of artificial intelligence in 3D bioprinting. Biophys. Rev..

[74] Groll J (2016). Biofabrication: reappraising the definition of an evolving field. Biofabrication.

[75] Lee J M, Ng W L, Yeong W Y (2019). Resolution and shape in bioprinting: strategizing towards complex tissue and organ printing. Appl. Phys. Rev..

[76] Filippou V, Tsoumpas C (2018). Recent advances on the development of phantoms using 3D printing for imaging with CT, MRI, PET, SPECT, and ultrasound. Med. Phys..

[77] Zaffino P, Moccia S, De Momi E, Spadea M F (2020). A review on advances in intra-operative imaging for surgery and therapy: imagining the operating room of the future. Ann. Biomed. Eng..

[78] Liu M, Drexler W (2019). Optical coherence tomography angiography and photoacoustic imaging in dermatology. Photochem. Photobiol. Sci..

[79] Hutchinson J C, Shelmerdine S C, Simcock I C, Sebire N J, Arthurs O J (2017). Early clinical applications for imaging at microscopic detail: microfocus computed tomography (micro-CT). Brit. J. Radiol..

[80] Zhu X, Menozzi L, Cho S-W, Yao J (2024). High speed innovations in photoacoustic microscopy. npj Imaging.

[81] Sexton Z A (2025). Rapid model-guided design of organ-scale synthetic vasculature for biomanufacturing. Science.

[82] Anon Advanced solutions-3D bioprinting & cutting-edge research for healthcare & life sciences. https://www.advancedsolutions.com/homepageasls.

[83] Anon Next generation bioprinter NGB: create complex 3D tissue models. https://www.poietis.com/.

[84] Zhu C, Gemeda H B, Duoss E B, Spadaccini C M (2024). Toward multiscale, multimaterial 3D printing. Adv. Mater..

[85] Liu S (2023). 3D Bioprinting tissue analogs: current development and translational implications. J. Tissue Eng..

[86] Ribezzi D (2023). Shaping synthetic multicellular and complex multimaterial tissues via embedded extrusion-volumetric printing of microgels. Adv. Mater..

[87] Größbacher G (2023). Volumetric printing across melt electrowritten scaffolds fabricates multi-material living constructs with tunable architecture and mechanics. Adv. Mater..

[88] Zhou H, Liu P, Gao Z, Li Q, Lv W, Yin J, Zhang B, Yang H, Ma L (2022). Simultaneous multimaterial multimethod bioprinting. Biodes. Manuf..

[89] Samandari M, Mostafavi A, Quint J, Memić A, Tamayol A (2022). *In situ* bioprinting: intraoperative implementation of regenerative medicine. Trends Biotechnol..

[90] Zhao W, Hu C, Xu T (2023). *In vivo* bioprinting: broadening the therapeutic horizon for tissue injuries. Bioact. Mater..

[91] Davoodi E (2025). Imaging-guided deep tissue *in vivo* sound printing. Science.

[92] Levin A A (2023). Commercial articulated collaborative *in situ* 3D bioprinter for skin wound healing. Int. J. Bioprint..

[93] Thai M T, Phan P T, Tran H A, Nguyen C C, Hoang T T, Davies J, Rnjak-Kovacina J, Phan H P, Lovell N H, Do T N (2023). Advanced soft robotic system for *in situ* 3D bioprinting and endoscopic surgery. Adv. Sci..

[94] Azizian M, Liu M, Khalaji I, Sorger J, Oh D, Daimios S (2020). The da vinci surgical system. Handbook of Robotic and Image-Guided Surgery.

[95] Kim J W (2025). SRT-H: a hierarchical framework for autonomous surgery via language-conditioned imitation learning. Sci. Robot..

[96] Martin I, Wendt D, Heberer M (2004). The role of bioreactors in tissue engineering. Trends Biotechnol..

[97] Kazimierczak P, Kalisz G, Sroka-Bartnicka A, Przekora A (2023). Effectiveness of the production of tissue-engineered living bone graft: a comparative study using perfusion and rotating bioreactor systems. Sci. Rep..

[98] Miller J S (2012). Rapid casting of patterned vascular networks for perfusable engineered three-dimensional tissues. Nat. Mater..

[99] Grayson W L, Fröhlich M, Yeager K, Bhumiratana S, Chan M E, Cannizzaro C, Wan L Q, Liu X S, Guo X E, Vunjak-Novakovic G (2010). Engineering anatomically shaped human bone grafts. Proc. Natl Acad. Sci. USA.

[100] Kolesky D B, Truby R L, Gladman A S, Busbee T A, Homan K A, Lewis J A (2014). 3D bioprinting of vascularized, heterogeneous cell-laden tissue constructs. Adv. Mater..

[101] Karbassi E, Fenix A, Marchiano S, Muraoka N, Nakamura K, Yang X, Murry C E (2020). Cardiomyocyte maturation: advances in knowledge and implications for regenerative medicine. Nat. Rev. Cardiol..

[102] Vining K H, Mooney D J (2017). Mechanical forces direct stem cell behaviour in development and regeneration. Nat. Rev. Mol. Cell Biol..

[103] Herland A (2020). Quantitative prediction of human pharmacokinetic responses to drugs via fluidically coupled vascularized organ chips. Nat. Biomed. Eng..

[104] Chung C, Burdick J A (2008). Engineering cartilage tissue. Adv. Drug Deliv. Rev..

[105] Zoneff E (2024). Controlled oxygen delivery to power tissue regeneration. Nat. Commun..

[106] Zhang B, Korolj A, Fook Lun Lai B, Radisic M (2018). Advances in organ-on-a-chip engineering. Nat. Rev. Mater..

[107] Ronaldson-Bouchard K, Przekwas A, Vunjak-Novakovic G (2022). A multi-organ chip with matured tissue niches linked by vascular flow. Nat. Biomed. Eng..

[108] Ingber D E (2022). Human organs-on-chips for disease modelling, drug development and personalized medicine. Nat. Rev. Genet..

[109] Lee K, Silva E A, Mooney D J (2011). Growth factor delivery-based tissue engineering: general approaches and a review of recent developments. J. R. Soc. Interface.

[110] Williams C G, Lee V J, Asatsuma T, Vento-Tormo R, Haque A (2022). An introduction to spatial transcriptomics for biomedical research. Genome Med..

[111] Ruta A, Krishnan K, Elisseeff J H (2024). Single-cell transcriptomics in tissue engineering and regenerative medicine. Nat. Rev. Bioeng..

[112] Argmann C (2023). Biopsy and blood-based molecular biomarker of inflammation in IBD. Gut.

[113] Wang Y, Fang L, Wang Y, Xiong Z (2023). Current trends of Raman spectroscopy in clinic settings: opportunities and challenges. Adv. Sci..

[114] Chang H, Hur W, Kang H, Jun B H (2025). *In vivo* surface-enhanced Raman scattering techniques: nanoprobes, instrumentation, and applications. Light Sci. Appl..

[115] Aebersold R, Mann M (2016). Mass-spectrometric exploration of proteome structure and function. Nature.

[116] Engvall E, Perlmann P (1971). Enzyme-linked immunosorbent assay (ELISA). Quantitative assay of immunoglobulin G. Immunochemistry.

[117] Chen F, Tillberg P W, Boyden E S (2015). Expansion microscopy. Science.

[118] Fernandez D C, Bhargava R, Hewitt S M, Levin I W (2005). Infrared spectroscopic imaging for histopathologic recognition. Nat. Biotechnol..

[119] Freudiger C W, Min W, Saar B G, Lu S, Holtom G R, He C, Tsai J C, Kang J X, Xie X S (2008). Label-free biomedical imaging with high sensitivity by stimulated Raman scattering microscopy. Science.

[120] Ozbolat I T, Hospodiuk M (2016). Current advances and future perspectives in extrusion-based bioprinting. Biomaterials.

[121] Kepp O, Galluzzi L, Lipinski M, Yuan J, Kroemer G (2011). Cell death assays for drug discovery. Nat. Rev. Drug Discov..

[122] Galluzzi L (2009). Guidelines for the use and interpretation of assays for monitoring cell death in higher eukaryotes. Cell Death Differ..

[123] Dowsett M (2011). Assessment of Ki67 in breast cancer: recommendations from the international Ki67 in breast cancer working group. J. Natl Cancer Inst..

[124] Gorgoulis V (2019). Cellular senescence: defining a path forward. Cell.

[125] Park Y, Depeursinge C, Popescu G (2018). Quantitative phase imaging in biomedicine. Nat. Photon..

[126] Zipfel W R, Williams R M, Webb W W (2003). Nonlinear magic: multiphoton microscopy in the biosciences. Nat. Biotechnol..

[127] Steinegger A, Wolfbeis O S, Borisov S M (2020). Optical sensing and imaging of pH values: spectroscopies, materials, and applications. Chem. Rev..

[128] Ates H C, Nguyen P Q, Gonzalez-Macia L, Morales-Narváez E, Güder F, Collins J J, Dincer C (2022). End-to-end design of wearable sensors. Nat. Rev. Mater..

[129] Teymourian H, Barfidokht A, Wang J (2020). Electrochemical glucose sensors in diabetes management: an updated review (2010–2020). Chem. Soc. Rev..

[130] Sebastian J A, Strohm E M, Baranger J, Villemain O, Kolios M C, Simmons C A (2023). Assessing engineered tissues and biomaterials using ultrasound imaging: *in vitro* and *in vivo* applications. Biomaterials.

[131] Kvåle Løvmo M, Deng S, Moser S, Leitgeb R, Drexler W, Ritsch-Marte M (2024). Ultrasound-induced reorientation for multi-angle optical coherence tomography. Nat. Commun..

[132] Koren K, Zieger S E (2021). Optode based chemical imaging—possibilities, challenges, and new avenues in multidimensional optical sensing. ACS Sens..

[133] Middya S, Curto V F, Fernández-Villegas A, Robbins M, Gurke J, Moonen E J M, Kaminski Schierle G S, Malliaras G G (2021). Microelectrode arrays for simultaneous electrophysiology and advanced optical microscopy. Adv. Sci..

[134] Park Y (2021). Three-dimensional, multifunctional neural interfaces for cortical spheroids and engineered assembloids. Sci. Adv..

[135] Zhang Y, Lee G, Li S, Hu Z, Zhao K, Rogers J A (2023). Advances in bioresorbable materials and electronics. Chem. Rev..

[136] Kang S K (2016). Bioresorbable silicon electronic sensors for the brain. Nature.

[137] Territo A (2025). Robot-assisted Kidney Transplantation: the 8-year European Experience. Eur. Urol..

[138] Prata F (2023). State of the art in robotic surgery with hugo RAS system: feasibility, safety and clinical applications. J. Pers. Med..

[139] Lazarou C, Moysidou E, Christodoulou M, Stai S, Lioulios G, Kasimatis E, Fylaktou A, Stangou M (2025). Protocol biopsies in kidney transplant recipients: current practice after much discussion. Biomedicines.

[140] Song Y (2025). Advancements in noninvasive techniques for transplant rejection: from biomarker detection to molecular imaging. J. Transl. Med..

[141] Holzhauser L, DeFilippis E M, Nikolova A, Byku M, Contreras J P, De Marco T, Hall S, Khush K K, Vest A R (2023). The end of endomyocardial biopsy?: a practical guide for noninvasive heart transplant rejection surveillance. Heart Fail..

[142] Oh K T, Mustehsan M H, Goldstein D J, Saeed O, Jorde U P, Patel S R (2021). Protocol endomyocardial biopsy beyond 6 months—it is time to move on. Am. J. Transplant..

[143] Madhvapathy S R (2023). Implantable bioelectronic systems for early detection of kidney transplant rejection. Science.

[144] Ertl P, Wladimir T, Sticker D, Schuller P, Rothbauer M, Wieselthaler G, Frauenlob M (2024). Development of a flexible sensor-integrated tissue patch to monitor early organ rejection processes using impedance spectroscopy. Biosensors.

[145] Knudsen J E, Ghaffar U, Ma R, Hung A J (2024). Clinical applications of artificial intelligence in robotic surgery. J. Robot. Surg..

[146] Kang H W, Lee S J, Ko I K, Kengla C, Yoo J J, Atala A (2016). A 3D bioprinting system to produce human-scale tissue constructs with structural integrity. Nat. Biotechnol..

[147] Lee V K, Kim D Y, Ngo H, Lee Y, Seo L, Yoo S S, Vincent P A, Dai G (2014). Creating perfused functional vascular channels using 3D bio-printing technology. Biomaterials.

[148] Hoffman T, Khademhosseini A, Langer R (2019). Chasing the paradigm: clinical translation of 25 years of tissue engineering. Tissue Eng. A.

[149] Briones Y, Pascua B, Tiangco N, Crisostomo I, Casiguran S, Remenyi R (2025). Assessing the landscape of clinical and observational trials involving bioprinting: a scoping review. 3D Print. Med..

[150] Anon NSW Health & Medical Research-Novel 3D bioprinting device ‘prints’ skin-growing ink from patient cells in world-first clinical trials. https://medicalresearch.nsw.gov.au/about/news/2025/novel-3d-bioprinting-device-prints-skin-growing-ink-from-patient-cells-in-world-first-clinical-trials.

[151] Dalton P D, Woodfield T B F, Mironov V, Groll J (2020). Advances in hybrid fabrication toward hierarchical tissue constructs. Adv. Sci..

[152] Bliley J M, Shiwarski D J, Feinberg A W (2022). 3D-bioprinted human tissue and the path toward clinical translation. Sci. Transl. Med..

[153] Agarwal T (2025). 3D bioprinting in tissue engineering: current state-of-the-art and challenges towards system standardization and clinical translation. Biofabrication.

[154] Arjmandmazidi S, Heidari H R, Ghasemnejad T, Mori Z, Molavi L, Meraji A, Kaghazchi S, Mehdizadeh Aghdam E, Montazersaheb S (2025). An in-depth overview of artificial intelligence (AI) tool utilization across diverse phases of organ transplantation. J. Transl. Med..

[155] De Kanter A F J, Jongsma K R, Verhaar M C, Bredenoord A L (2023). The ethical implications of tissue engineering for regenerative purposes: a systematic review. Tissue Eng. B.

[156] Otto I A, Breugem C C, Malda J, Bredenoord A L (2016). Ethical considerations in the translation of regenerative biofabrication technologies into clinic and society. Biofabrication.

[157] Horch R E, Popescu L M, Vacanti C, Maio G (2008). Ethical issues in cellular and molecular medicine and tissue engineering. J. Cell Mol. Med..

[158] Kirillova A, Bushev S, Abubakirov A, Sukikh G (2020). Bioethical and legal issues in 3D bioprinting. Int. J. Bioprint..

[159] Bohr A, Memarzadeh K (2020). The rise of artificial intelligence in healthcare applications. Artificial Intelligence Healthcare.

[160] Char D S, Abràmoff M D, Feudtner C (2020). Identifying ethical considerations for machine learning healthcare applications. Am. J. Bioethics.

[161] Wheeler N E (2025). Responsible AI in biotechnology: balancing discovery, innovation and biosecurity risks. Front. Bioeng. Biotechnol..

[162] Snyder D (2025). Pioneering ethical oversight in AI-enabled clinical research: insights on the new framework | WCG. https://www.centerwatch.com/insights/pioneering-ethical-oversight-in-ai-enabled-clinical-research-insights-on-the-new-framework/.

[163] Frenette J (2023). Ensuring human oversight in high-performance AI systems: a framework for control and accountability. World J. Adv. Res. Rev..

[164] London A J (2019). Artificial intelligence and black-box medical decisions: accuracy versus explainability. Hastings Cent. Rep..

[165] Gerke S, Minssen T, Cohen G (2020). Ethical and legal challenges of artificial intelligence-driven healthcare. Artificial Intelligence Healthcare.

[166] Li F, Ruijs N, Lu Y (2022). Ethics & AI: a systematic review on ethical concerns and related strategies for designing with AI in healthcare. Ai.

[167] Buhmann A, Fieseler C (2021). Towards a deliberative framework for responsible innovation in artificial intelligence. Technol. Soc..

[168] Gerardi C, Xinaris C (2025). Beyond human limits: the ethical, social, and regulatory implications of human enhancement. Front. Med. Lausanne.

[169] Gilman M (2023). Data & society—democratizing AI principles for meaningful public participation. https://digitalgovernmenthub.org/library/democratizing-ai-principles-for-meaningful-public-participation/.

